# The Use of the Clinical Global Impressions Scale in Psychosocial Interventions for Autism Characteristics: A Systematic Review

**DOI:** 10.1177/13623613261472939

**Published:** 2026-08-09

**Authors:** Magdalena Glod, Victoria Grahame, Jacqui Rodgers

**Affiliations:** 1 Newcastle University, Newcastle upon Tyne, UK; 2 Walkergate Park Centre for Neurorehabilitation and Neuropsychiatry, Newcastle upon Tyne, UK; 3 Royal Victoria Infirmary, Newcastle upon Tyne, UK

**Keywords:** autism spectrum disorder, Clinical Global Impressions (CGI) scale, psychosocial interventions, systematic review

## Abstract

The increase in intervention diversity to improve core difficulties in autism has resulted in the development and proliferation of a variety of measures to assess change. The Clinical Global Impressions (CGI) scale has been recommended for use in pharmacological autism clinical trials to monitor individuals’ progress. No specific recommendations have been made for an outcome measure for psychosocial interventions. This review aimed to evaluate available evidence of a use of the CGI scale as an outcome measure in psychosocial interventions for autism characteristics. A systematic search of electronic databases identified 22 primary studies utilising the CGI scale to evaluate change across a range of social, communication and behavioural interventions for autistic people. The lack of consistency in the use of the measure was evident. This applied to the variety of constructs being measured, inconsistent application and scoring methods, which all limited the CGI scale’s reliability and validity. Despite the widespread use of the CGI scale in psychosocial clinical trials for autistic people, the lack of standardisation hampers conclusions regarding the evidence of its effectiveness in measuring change. Standardisation of the use of the CGI scale is essential to ensure comprehensive evaluation of interventions.

**Lay Abstract:**

Many different therapies and supports exist to help autistic people with social, communication, and behavioural differences. To understand whether these interventions are working, researchers need good tools to measure change. One commonly used tool in medical studies is the Clinical Global Impressions (CGI) scale, which helps clinicians rate a person’s overall improvement. Although the CGI scale is recommended for autism medication studies, there are no clear guidelines for using it in psychosocial (non-medical) interventions. In this review, we looked at research studies that used the CGI scale to measure change after psychosocial interventions for autistic people. We found 22 studies that used the CGI scale in very different ways. The studies measured a wide range of skills and behaviours, and they often used the CGI scale differently from one another. This inconsistency makes it difficult to trust the results or compare findings across studies. Although the CGI scale is widely used, the lack of standard guidelines means it may not reliably show whether psychosocial interventions are effective. To improve research and ensure that interventions are properly evaluated, it is important to develop clear, standardised ways of using the CGI scale.

## Introduction

Autism spectrum disorder (ASD; hereafter referred to as autism) is a lifelong neurodevelopmental condition characterised by persistent difficulties in social communication and interaction, as well as restricted, repetitive patterns of behaviour and atypical sensory responses (American Psychiatric Association [APA], *DSM-5* Task Force, 2013). The prevalence of autism has increased substantially in recent decades, with a 787% rise in diagnoses reported in the United Kingdom between 1998 and 2018 (Russell et al., 2022) and current estimates suggesting it affects approximately one in 31 children in the United States (Shaw et al., 2025). As prevalence has grown, so too has the need for effective services and interventions to support autistic people with their complex needs (de Nocker & Toolan, 2023).

A wide range of interventions have been developed to address the core characteristics of autism, such as social communication, language, and play skills (Bishop-Fitzpatrick et al., 2013; Sandbank et al., 2020), as well as associated mental health difficulties including anxiety (Delli et al., 2018; South et al., 2017). The growing diversity of interventions has led to an expansion of tools used to measure change following treatment (Provenzani et al., 2020). Currently, there is no consensus on a standardised battery of measures to assess core changes or intervention response in autism (Brugha et al., 2015; Magiati et al., 2011) yet reliable and valid tools are essential for evaluating treatment outcomes and guiding evidence-based clinical practice (Brugha et al., 2015).

The Clinical Global Impressions (CGI; Guy, 1976) scale is a brief, clinician-rated tool widely used to monitor patient progress in pharmacological trials (Toolan et al., 2022). The complete CGI scale consists of three different global items that rate: (a) Severity of Illness (CGI-S), (b) Global Improvement (CGI-I), and (c) the Efficacy Index evaluating the balance between therapeutic effects and side effects (Guy, 1976). The first two scales, CGI-Severity (CGI-S) and CGI-Improvement (CGI-I), contain a single question that is rated by a clinician on a 7-point scale, ranging from 1 = *normal, not at all ill* to 7 = *among the most extremely ill patients* for the CGI-S and from 1 = *very much improved* to 7 = *very much worse* for the CGI-I (Guy, 1976). The CGI-S is typically used at baseline and endpoint, whereas the CGI-I is administered during and after treatment to assess change (Aman et al., 2004). The CGI has demonstrated acceptable inter-rater and test–retest reliability (Busner & Targum, 2007; Zaheer et al., 2013) and good construct validity (Kadouri et al., 2007).

Although the CGI is widely used in pharmacological trials for autism (Aman et al., 2004; Farmer et al., 2013), it has also been applied in psychosocial interventions, including those targeting social communication and behavioural change (Bearss et al., 2015; Guastella et al., 2015; Storch et al., 2013). However, to date, there has been no systematic review examining the use and utility of the CGI scale in psychosocial interventions for autism characteristics.

This review therefore aims to provide a systematic summary and critical evaluation of existing evidence, identify research gaps, and offer recommendations for future research and clinical practice. Specifically, it seeks to answer the following question: How has the CGI (Guy, 1976) scale been used as an outcome measure in psychosocial interventions for autism characteristics?

## Method

### Protocol and Registration

The review protocol was registered online with the International Prospective Register of Systematic Reviews (Registration number: CRD42024597571) and can be accessed at http://www.crd.york.ac.uk/PROSPERO/prospero.asp.

### Search Strategy

A systematic electronic search was conducted to identify original studies that utilised the CGI scale in psychosocial interventions for autism characteristics. Six databases, Scopus, Web of Knowledge, APA PsycINFO, Embase, Medline, and the Cochrane Library, were searched for studies published between January 2000 and January 2025. The start date corresponds to the release of the *Diagnostic and Statistical Manual of Mental Disorders* (4th ed., text rev.; *DSM-IV-TR*; APA, 2000).

Search terms were designed to capture both autism-related diagnostic terminology and the use of the CGI scale. In Scopus and Web of Knowledge, the following terms were applied: autis OR Asperger* OR ASD OR “pervasive developmental disorder*” OR PDD*) AND “clinical global impression*” OR CGI-I OR CGII OR CGI-S OR CGIS. In APA PsycINFO, Embase, Medline, and the Cochrane Library, controlled vocabulary terms were used in accordance with each database’s indexing system (e.g., MeSH for Medline, APA Thesaurus for PsycINFO). General keywords were added alongside controlled vocabulary in all databases.

Diagnostic terms were limited to titles, abstracts, and keywords, whereas CGI terms were searched across all available fields. The abbreviation CGI alone was excluded to avoid retrieving records related to “computer-generated imagery.” All search results were imported into EndNote for reference management.

A summary of the search strategy and the number of records identified, screened, and excluded at each stage is presented in Figure 1.

**Figure 1.**
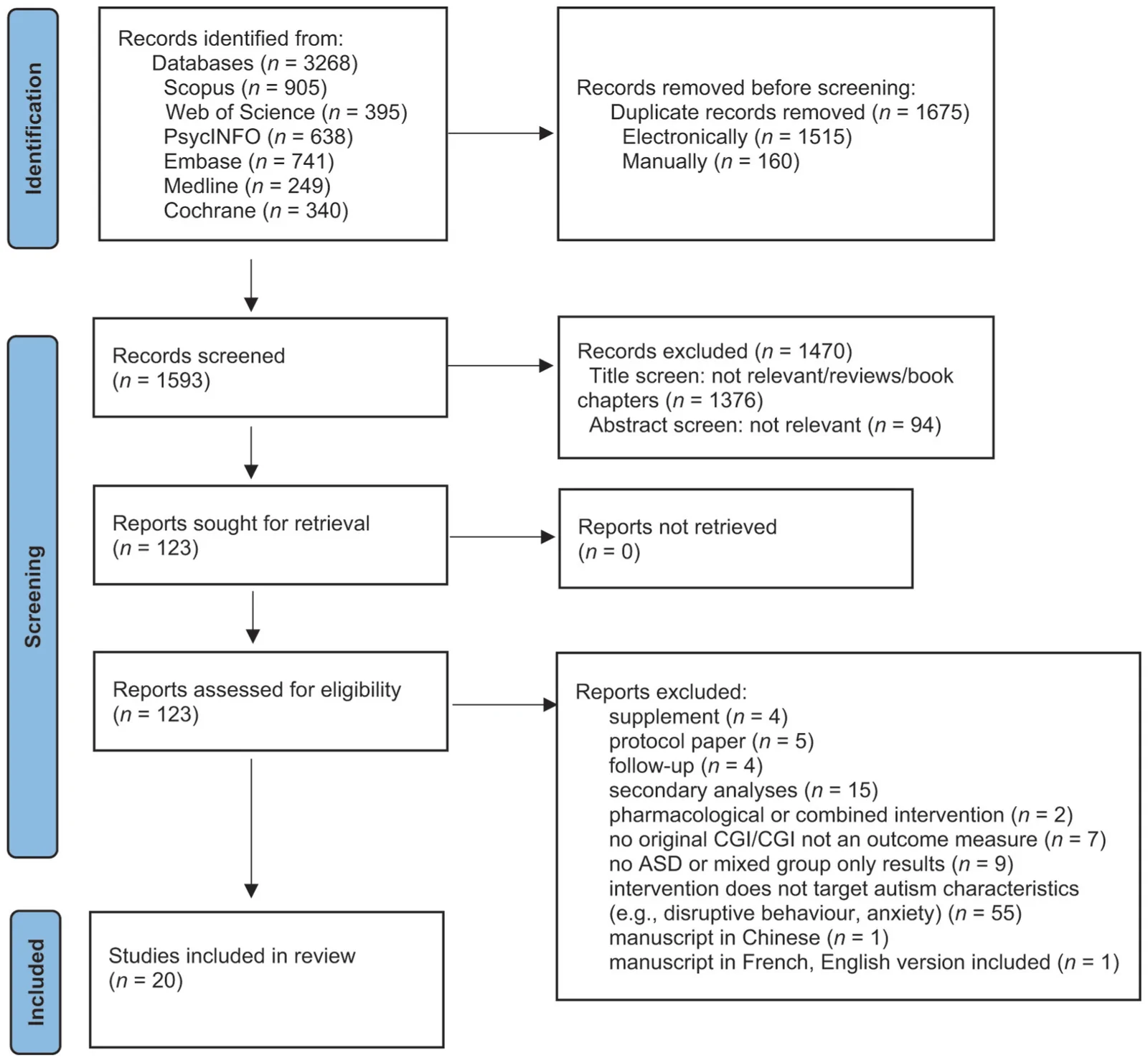
PRISMA flow diagram showing the identification and selection procedure of included articles (Page et al., 2021).

### Selection Criteria

The inclusion and exclusion criteria were defined before the literature search was conducted. Studies that met the following criteria were included in the review: (a) original articles published in peer-reviewed scientific journals; (b) studies involving individuals with an autism/ASD diagnosis; (c) when mixed samples were used, data were presented separately for the autistic group; (d) inclusion of a psychosocial intervention (direct or indirect, e.g., aimed at parents or teachers) targeting autism characteristics as per the *DSM-5* (APA, 2013); (e) studies using either quasi-experimental or experimental designs, including single-case experimental designs; and (f) studies using the original CGI scale (Guy, 1976) as an outcome measure.

The following were excluded: non-primary research (e.g., reviews, books), pharmacological interventions for autism symptoms (e.g., selective serotonin reuptake inhibitors, stimulants, and α2-adrenergic agonists), as well as complementary and alternative medicine (CAM) approaches (Zollman & Vickers, 1999). Accordingly, both non-biological interventions (e.g., craniosacral manipulation, equine-assisted therapy) and biological treatments (e.g., immunoregulatory, dietary supplement) were excluded (for reviews of pharmacological and CAM treatments, see Huffman et al., 2011).

Protocol papers, follow-up studies, and secondary analyses were also excluded. No restrictions were applied regarding participant age. A summary of inclusion and exclusion criteria is presented in Table 1.

**Table 1.** The Summary of the Inclusion and Exclusion Criteria.

Criteria	Inclusion	Exclusion
Study Design	• Quasi-experimental designs • Experimental designs	• Reviews • Meta-analyses
Population	• individuals with an autism/ASD diagnosis • Mixed samples – data presented separately for the autistic group	• * individuals with no autism/ASD diagnosis
Intervention/exposure	• Psychosocial interventions targeting autism characteristics as per the *DSM-5* (APA, 2013)	• Pharmacological treatments for autism symptoms • Complementary and alternative medicine treatments • Interventions targeting other difficulties
Outcomes	• CGI scale (Guy, 1976) used as an outcome measure	• Outcomes not relevant to the research question
Language	• English • Other languages	• Not applicable
Publication type	• Original, peer-reviewed journal articles	• Protocol papers • Follow-up studies • Secondary analyses • Book chapters • Conference abstracts
Timeframe	• Studies published from the year 2000	• Studies published before the year 2000

One reviewer screened all titles and abstracts, with any uncertainties retained for full-text review. Full-text screening was performed by two reviewers against the inclusion criteria, achieving 91% inter-rater agreement. Ambiguous papers (*n* = 11) were further discussed until consensus was reached.

### Final Selection

One hundred twenty-three eligible records were full-text screened. One hundred three papers were excluded. Four papers were excluded due to unpublished status (conference papers). Five papers were protocol papers, four were follow-up papers, and 15 papers focused on secondary analyses, whereas the original interventions were reported elsewhere. Two papers investigated pharmacological or combined interventions. Another six papers did not utilise the original CGI (e.g., the Ohio Autism Clinical Impressions Scale [OACIS] and the OSU Autism CGI scale were used, see the Discussion section) and one study used the CGI scale as a measure of the intervention and not an outcome (Kasari et al., 2021). Eight studies either did not include autistic participants or did not report results separately for the autistic subgroup, whereas 55 focused on co-occurring conditions rather than autism characteristics (e.g., emotion dysregulation, disruptive behaviour, anxiety). One manuscript was available only in Chinese, and one duplicate French version was excluded. Twenty papers were included in the systematic review.

### Complementary Search

A complementary literature search was also performed. Similar to the main search, it was extended from January 2000 to January 2025. This search, however, focused on identifying autism studies that cited Guy (1976) to identify manuscripts that utilised the CGI in psychosocial interventions for autism characteristics. Four databases (Scopus, Web of Knowledge, APA PsycINFO, and Medline) allowed reference searches. Autism-related search terms consistent with the main search were applied to titles, abstracts, and keywords. The reference field was filtered with database-specific alteration of “ECDEU assessment manual for psychopharmacology” and specific field abbreviations (e.g., .rf in Medline and .cv in PsycINFO). Identified records were imported into Endnote. A flowchart of the complementary search strategy is presented in Figure 2.

**Figure 2.**
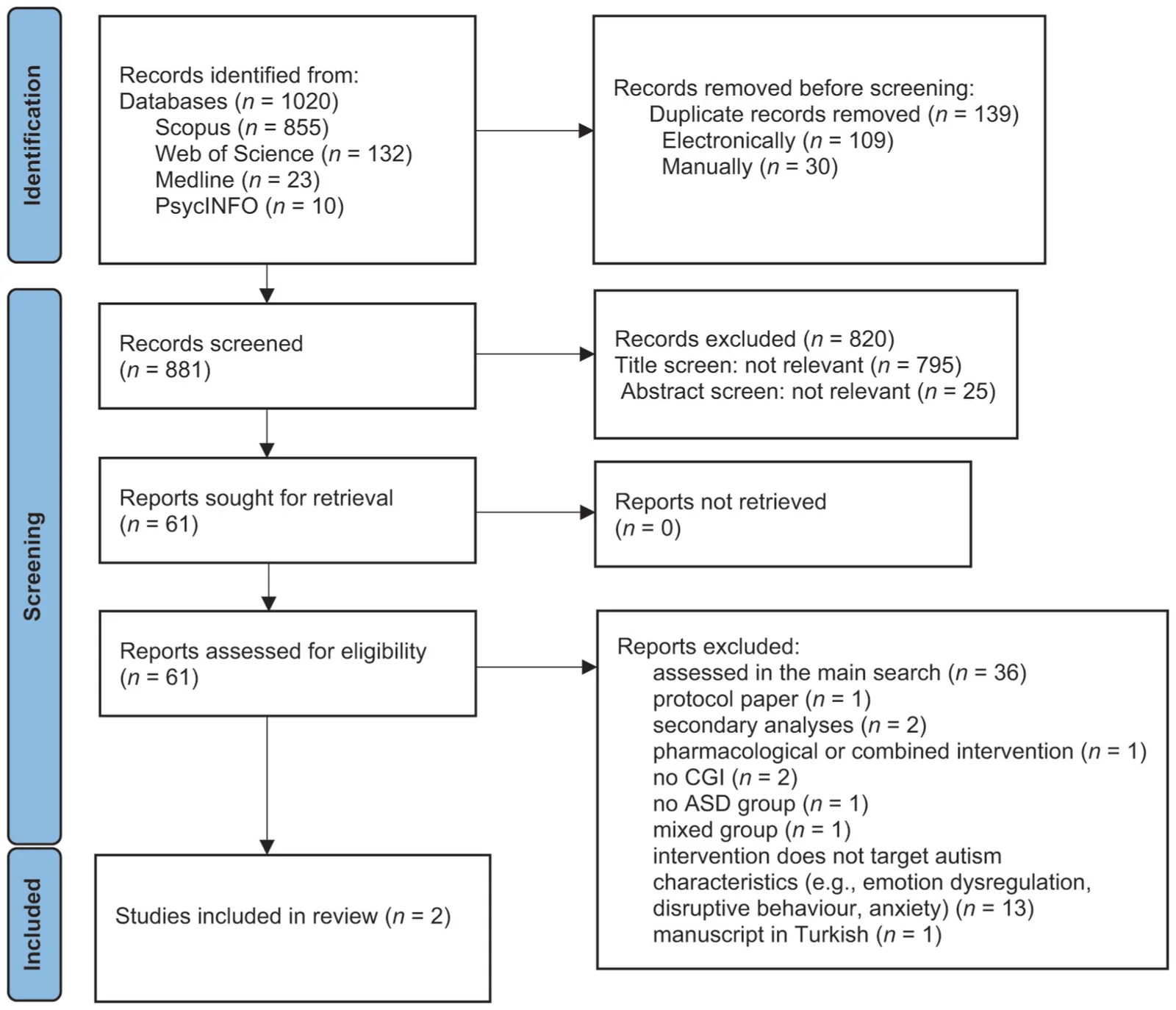
PRISMA flow diagram showing the identification and selection procedure of included articles in the complementary literature search (Page et al., 2021).

### Complementary Search – Final Selection

Full-text screening was conducted for 61 papers. Fifty-nine papers were excluded after full-text screening. Most had already been assessed in the main search, with 36 duplicates. Of the remaining 23 manuscripts, exclusions were as follows: One protocol paper, two reporting secondary analyses, one investigating pharmacological or combined interventions, one lacking autistic participants, one reporting only mixed-group results, two not using the original CGI, and one available only in Turkish. Thirteen studies targeted co-occurring conditions rather than core autism characteristics.

Following these exclusions, two additional studies met the inclusion criteria and were incorporated into the review.

### Quality Assessment and Risk-of-Bias Evaluation

Quality assessment and risk-of-bias evaluation were conducted for all included studies. For randomised controlled trials (RCTs), the Cochrane Risk-of-Bias 2 tool (RoB 2) for individually randomised parallel-group trials (Sterne et al., 2019) was used to assess potential biases across selection, performance, detection, and reporting domains. Single-arm studies were evaluated using the Joanna Briggs Institute critical appraisal tool (Munn et al., 2020), which considers methodological quality, statistical analysis, and reporting of results. These tools identify potential sources of bias, assess the overall strength of evidence, and inform the reliability of review findings.

As subjective judgement is inherent in these assessments (Haddaway et al., 2015; Nashwan & Jaradat, 2023), a subset of studies (*n* = 6; 27%) was independently evaluated by two raters. Interrater reliability was calculated, with 83.3% agreement across checklist criteria.

## Results

A total of 22 papers met the inclusion criteria and were retained for review. For each study, information was extracted on the first author, year of publication, country of origin, intervention name and aim, study aim and design, sample characteristics (gender, sample size, age, diagnosis), and main findings (see Supplemental Appendix A for a summary of the descriptive characteristics of the included studies).

### Interventions

Across the 22 studies using the CGI scale (Guy, 1976) as an outcome measure, a variety of psychosocial interventions targeting autism characteristics were implemented. The majority focused on improving social and communication skills. Five studies evaluated Pivotal Response Treatment (PRT), delivered by clinicians (de Korte et al., 2021; Ventola et al., 2014), parents (Hardan et al., 2015), a combination of parent training and clinician-delivered intervention (Gengoux, Abrams, et al., 2019), or with and without robot assistance (van den Berk-Smeekens et al., 2022). Two studies implemented the Early Start Denver Model (ESDM) in individual (Tateno et al., 2021) or group settings (Zitter et al., 2023). Two studies assessed music therapy (Freire et al., 2021; Rabeyron et al., 2020), and one applied a developmental approach, Developmental Reciprocity Treatment (DTR) (Gengoux, Schapp, et al., 2019). Three studies used cognitive-behavioural approaches: Eye Movement Desensitization and Reprocessing (EMDR; Leuning et al., 2023), the cognitive-behavioural summer treatment (summerMAXyc; Thomeer et al., 2020), and the Multimodal Anxiety and Social Skills Intervention (MASSI; White et al., 2013). Interventions targeting social skills included tabletop role-playing games (TTRPGs; Henning et al., 2024), College and Living Success (CLS), and Brain-Computer Interface for ASD (BCI-ASD; White et al., 2016), a modified UCLA PEERS programme (Sittanomai et al., 2021), Systematic Use of Cooperative Contingencies to Enhance Social Success (SUCCESS; Shkel et al., 2025), and Social Initiation Motivation Intervention (SIMI; Gengoux et al., 2021). Parent-mediated interventions included training to stimulate joint attention and language skills (Oosterling et al., 2010) and language-focused interventions (Scahill et al., 2022).

Finally, two studies targeted restricted and repetitive behaviours in autistic children (Grahame et al., 2015; Shiri et al., 2020).

### Participants

Across the 22 included studies, a total of 734 autistic children and adolescents participated in psychosocial interventions, with sample sizes ranging from five (Zitter et al., 2023) to 83 participants (Scahill et al., 2022; van den Berk-Smeekens et al., 2022). Participant ages ranged from 15 months to 25 years. Four studies focused on very early interventions for toddlers (Oosterling et al., 2010; Shiri et al., 2020; Tateno et al., 2021; Zitter et al., 2023), whereas several targeted young and middle-aged children (de Korte et al., 2021; Freire et al., 2021; Gengoux, Abrams et al., 2019; Gengoux et al., 2021; Gengoux, Schapp et al., 2019; Grahame et al., 2015; Hardan et al., 2015; Rabeyron et al., 2020; Scahill et al., 2022; Shkel et al., 2025; Thomeer et al., 2020; van den Berk-Smeekens et al., 2022; Ventola et al., 2014). Three studies focused on teenagers (Henning et al., 2024; Leuning et al., 2023; White et al., 2013), and one study included young adults (White et al., 2016).

Gender was reported in all studies, with 609 males (83%), reflecting the typically observed male predominance in autism (Anello et al., 2009), although higher than the more recently suggested 3:1 male-to-female ratio (Loomes et al., 2017).

Regarding cognitive ability and co-occurring conditions, seven studies included participants with autism without specifying cognitive status or co-occurring difficulties (Freire et al., 2021; Gengoux, Schapp, et al., 2019; Rabeyron et al., 2020; Shiri et al., 2020; Tateno et al., 2021; White et al., 2016; Zitter et al., 2023). Nine studies excluded individuals with intellectual disability (ID), either by specifying a minimum IQ (⩾70 or ⩾80; (de Korte et al., 2021; Gengoux et al., 2021; Leuning et al., 2023; Shkel et al., 2025; Sittanomai et al., 2021; van den Berk-Smeekens et al., 2022), referring to high-functioning autism (Thomeer et al., 2020), cognitively-able autistic individuals (Ventola et al., 2014) or moderate/severe autism exclusions (Henning et al., 2024). One study included autistic children and those with pervasive developmental disorders with a developmental age above 12 months (Oosterling et al., 2010), and only Grahame et al. (2015) explicitly included children across a range of abilities. Three studies focused on participants with significant language or communication delays (Gengoux, Abrams, et al., 2019; Hardan et al., 2015; Scahill et al., 2022), whereas White et al. (2013) included only autistic individuals with a comorbid anxiety disorder.

### Study Design

Most studies using the CGI as an outcome measure in psychosocial interventions for autism characteristics were RCTs (de Korte et al., 2021; Freire et al., 2021; Gengoux et al., 2021; Gengoux, Abrams, et al., 2019; Grahame et al., 2015; Hardan et al., 2015; Oosterling et al., 2010; Rabeyron et al., 2020; Scahill et al., 2022; Shkel et al., 2025; van den Berk-Smeekens et al., 2022; White et al., 2013, 2016). The remaining nine were single-arm studies, either described as pre–post designs or without explicit labelling (Gengoux, Schapp, et al., 2019; Henning et al., 2024; Leuning et al., 2023; Shiri et al., 2020; Sittanomai et al., 2021; Tateno et al., 2021; Thomeer et al., 2020; Ventola et al., 2014; Zitter et al., 2023). Findings related to the CGI scale, such as components informing scoring, blinding, and effect sizes (see Supplemental Appendices B and C) were synthesised within these two research designs, reflecting the evidence hierarchy, with RCTs at the top and single-arm studies at the lower level (Burns et al., 2011).

### CGI as an Outcome Measure

All 13 RCTs used the CGI-I as an outcome measure. Three of these selected the CGI-I as their primary outcome measure (Grahame et al., 2015; Rabeyron et al., 2020; van den Berk‑Smeekens et al., 2022), whereas seven studies stated that the CGI-I was a secondary outcome measure (de Korte et al., 2021; Gengoux, Abrams, et al., 2019; Gengoux et al., 2021; Hardan et al., 2015; Oosterling et al., 2010; Scahill et al., 2022; White et al., 2013). The remaining RCTs did not specify its status. One RCT used the CGI-S only for baseline characterisation (de Korte et al., 2021), whereas three also included it as an outcome measure, either as a secondary outcome measure (Gengoux, Abrams, et al., 2019; Hardan et al., 2015) or as an unspecified outcome measure (Freire et al., 2021).

Eight single-arm studies implemented the CGI-I as an outcome measure, Thomeer et al. (2020) selected it as a primary outcome measure, whereas Leuning et al. (2023) as a secondary outcome measure. The other single-arm studies did not specify whether the CGI-I was a primary or secondary outcome measure. In three single-arm studies, the CGI-S was also used as an outcome measure (Gengoux, Schapp, et al., 2019; Henning et al., 2024; Leuning et al., 2023). One study selected the CGI-S as an outcome measure (Tateno et al., 2021).

### What Was the CGI Used as an Assessment of?

Studies described various constructs assessed by the CGI. Change in global functioning or overall improvement was explicitly reported in six RCTs (de Korte et al., 2021; Oosterling et al., 2010; Scahill et al., 2022; van den Berk-Smeekens et al., 2022; White et al., 2013, 2016) and four single-arm studies (Leuning et al., 2023; Shiri et al., 2020; Sittanomai et al., 2021; Zitter et al., 2023). Some studies used alternative terms, such as general behaviour and performance (Freire et al., 2021), overall severity of autism symptoms, behaviour, and function (Tateno et al., 2021), or overall clinical effect (Ventola et al., 2014). Assessing severity was noted by Rabeyron et al. (2020). Several studies across designs used the CGI to measure social communication (Gengoux, Abrams, et al., 2019; Gengoux et al., 2021; Gengoux, Schapp et al., 2019; Hardan et al., 2015; Henning et al., 2024; Shkel et al., 2025; Thomeer et al., 2020) or overall improvement in parent–child interaction (Grahame et al., 2015).

### CGI Components Included to Inform the CGI Scoring

Across studies, various assessment components informed CGI scoring, including clinician interviews (de Korte et al., 2021; van den Berk-Smeekens et al., 2022), parent interviews (Freire et al., 2021), video recordings with parent interviews (Gengoux et al., 2021; Shkel et al., 2025), questionnaires, clinical measures, vignettes, and interaction videos (Grahame et al., 2015), assessment reports (Oosterling et al., 2010), vignettes and questionnaires (Scahill et al., 2022), parent and child interviews (White et al., 2013), and self-reports, interviews, and observations (White et al., 2016). Three RCTs did not specify the information used for CGI scoring (Gengoux, Abrams, et al., 2019; Hardan et al., 2015; Rabeyron et al., 2020). Similarly, single-arm studies used parent interviews (Gengoux, Schapp, et al., 2019), clinician interviews (Leuning et al., 2023), questionnaires and observations (Shiri et al., 2020), video recordings (Thomeer et al., 2020), record reviews (Ventola et al., 2014), and questionnaires (Zitter et al., 2023). Three did not report their CGI scoring basis (Henning et al., 2024; Sittanomai et al., 2021; Tateno et al., 2021).

### Rater

In most studies, clinicians completed the CGI rating – nine RCTs (de Korte et al., 2021; Gengoux et al., 2021; Grahame et al., 2015; Hardan et al., 2015; Oosterling et al., 2010; Rabeyron et al., 2020; Shkel et al., 2025; van den Berk-Smeekens et al., 2022; White et al., 2016) and five single-arm studies (Gengoux, Schapp, et al., 2019; Leuning et al., 2023; Sittanomai et al., 2021; Ventola et al., 2014; Zitter et al., 2023). Research staff conducted ratings in one RCT (Gengoux, Abrams, et al., 2019) and one single-arm study (Tateno et al., 2021). Independent evaluators were reported in two RCTs (Scahill et al., 2022; White et al., 2013) and one single-arm study (Thomeer et al., 2020). Parents or carers completed the CGI in two single-arm studies (Henning et al., 2024; Tateno et al., 2021). One RCT did not specify the rater (Freire et al., 2021), and the description in Shiri et al. (2020) was unclear, citing both clinicians and an independent evaluator.

### Blinding

CGI raters were blinded to treatment allocation in 12 of the 13 RCTs; only Freire et al. (2021) did not report on blinding. In five single-arm studies, assessors were blinded to study aims (Leuning et al., 2023; Shiri et al., 2020; Thomeer et al., 2020; Ventola et al., 2014; Zitter et al., 2023). One single-arm study did not specify blinding status (Tateno et al., 2021), and the remaining three did not implement blinding for CGI evaluators.

### Inter-Rater Reliability

Only two RCTs reported inter-rater reliability for CGI ratings. White et al. (2013) described reliability training using case vignettes, whereas White et al. (2016) noted assessor training to a predefined reliability standard. In the remaining RCTs, reliability was either not calculable (single rater) or not reported. Among single-arm studies, one reported absolute agreement between two raters (Thomeer et al., 2020), and two referred to reliability training (Ventola et al., 2014; Zitter et al., 2023).

### CGI Timepoints

CGI assessment time points varied widely across studies, reflecting intervention duration, mid-point assessments, and follow-up periods. Ratings were completed as early as 6 weeks after intervention onset (Hardan et al., 2015) and up to 48 weeks of post-treatment follow-up (Scahill et al., 2022). Weeks 10 and 12 were the most common CGI assessment points (e.g., van den Berk-Smeekens et al., 2022; Shkel et al., 2025).

### CGI Scoring and Interpretation

Nine RCTs described using a 7-point Likert-type scale for the CGI (de Korte et al., 2021; Gengoux et al., 2021; Grahame et al., 2015; Oosterling et al., 2010; Scahill et al., 2022; Shkel et al., 2025; van den Berk-Smeekens et al., 2022; White et al., 2013, 2016). Rabeyron et al. (2020) referred to the CGI-S 7-point scale while implementing the CGI-I. The remaining RCTs did not specify scoring details. All single-arm studies reported using the 7-point scale, although Ventola et al. (2014) applied modified anchor points.

Seven RCTs (de Korte et al., 2021; Grahame et al., 2015; Rabeyron et al., 2020; Scahill et al., 2022 van den (Berk-Smeekens et al., 2022; White et al., 2013, 2016) and three single-arm studies (Leuning et al., 2023; Shiri et al., 2020; Thomeer et al., 2020) provided clinical interpretations of CGI scores when used as categorical outcomes. Rabeyron et al. (2020) defined “clinically meaningful improvement” as a ⩾2-point decrease. Terms such as “clinical responders” (de Korte et al., 2021; Leuning et al., 2023; van den Berk-Smeekens et al., 2022), “improvement” (Grahame et al., 2015), “treatment responders” (White et al., 2016), “positive response” (Scahill et al., 2022), and “responders” (Shiri et al., 2020; Thomeer et al., 2020; White et al., 2013) were typically defined as a score of 1 (*very much improved*) or 2 (*much improved*) on the CGI scale, with White et al. (2016) also including a score of 3 (*minimally improved*).

### Power Calculation

Only six RCTs reported sample size and power calculations (de Korte et al., 2021; Gengoux, Abrams, et al., 2019; Hardan et al., 2015; Oosterling et al., 2010; Scahill et al., 2022; White et al., 2013). Of these, only de Korte et al. (2021) recruited the full sample needed to achieve adequate power, whereas Scahill et al. (2022) performed a *post hoc* power calculation due to under-recruitment.

### Results – p Values, Effect Sizes, and Other Comparisons

Significant group differences on the CGI were reported in eight RCTs (Freire et al., 2021; Gengoux, Abrams, et al., 2019; Gengoux et al., 2021; Grahame et al., 2015; Hardan et al., 2015; Rabeyron et al., 2020; Scahill et al., 2022; Shkel et al., 2025), while the remaining RCTs found no significant differences. Effect sizes, either reported or calculated, were large for CGI-I (de Korte et al., 2021; Freire et al., 2021; Gengoux, Abrams, et al., 2019; Hardan et al., 2015) and medium for CGI-S (Gengoux, Abrams, et al., 2019; Hardan et al., 2015), always favouring the intervention.

Similarly, four single-arm studies reported significant pre–post differences on the CGI (Gengoux, Schapp, et al., 2019; Leuning et al., 2023; Tateno et al., 2021; Thomeer et al., 2020). Effect sizes were large for CGI-I (Thomeer et al., 2020) and medium-to-large for CGI-S (Gengoux, Schapp, et al., 2019; Leuning et al., 2023; Tateno et al., 2021).

Several studies reported the proportion of participants classified as responders on the CGI. These ranged from 64% responders in the treatment group versus none in the waitlist group (Shkel et al., 2025) to no difference between conditions (White et al., 2016).

### Risk of Bias

Of the 22 autism intervention studies included, most had small sample sizes, ranging from five (Zitter et al., 2023) to 83 participants (Scahill et al., 2022; van den Berk-Smeekens et al., 2022), and several lacked a comparison group. Among the 13 RCTs, only four (29%; Gengoux et al., 2021; Hardan et al., 2015; Scahill et al., 2022; White et al., 2013) were rated as low risk of bias across all Cochrane RoB 2 domains (Sterne et al., 2019; Figure 3). Common concerns in the remaining RCTs included lack of blinding for carers and intervention providers and incomplete reporting of deviations from intended interventions. All single-arm studies showed methodological concerns per the Joanna Briggs Institute tool (Munn et al., 2020; Figure 4), mainly selective participant inclusion and limited clinical information. Only two studies (White et al., 2013, 2016) reported reliability training for CGI assessors.

**Figure 3.**
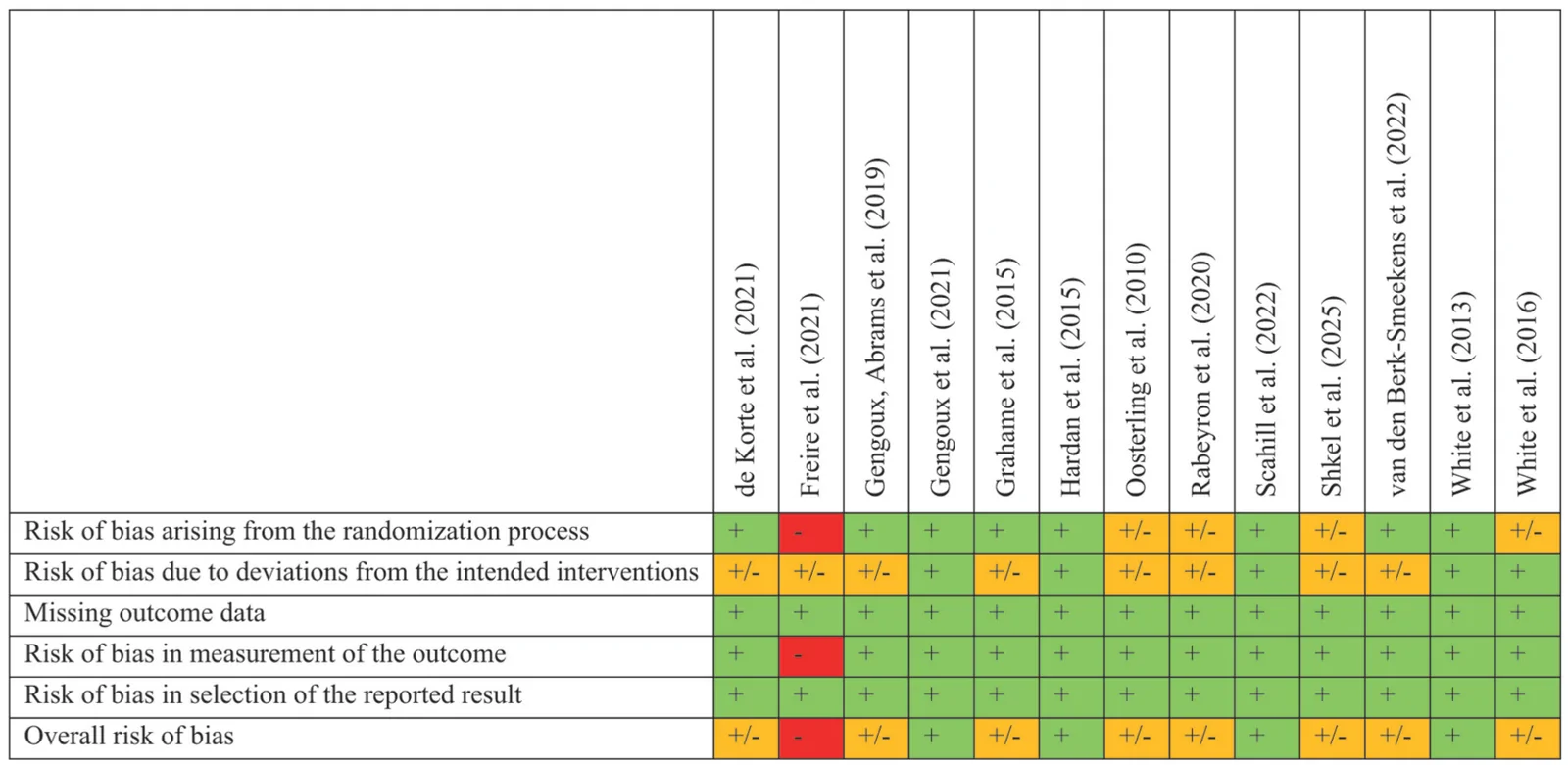
Cochrane Risk-of-Bias 2 tool, table of results. *Note*. Low risk of bias +, Some concerns +/−, High risk of bias –.

**Figure 4.**
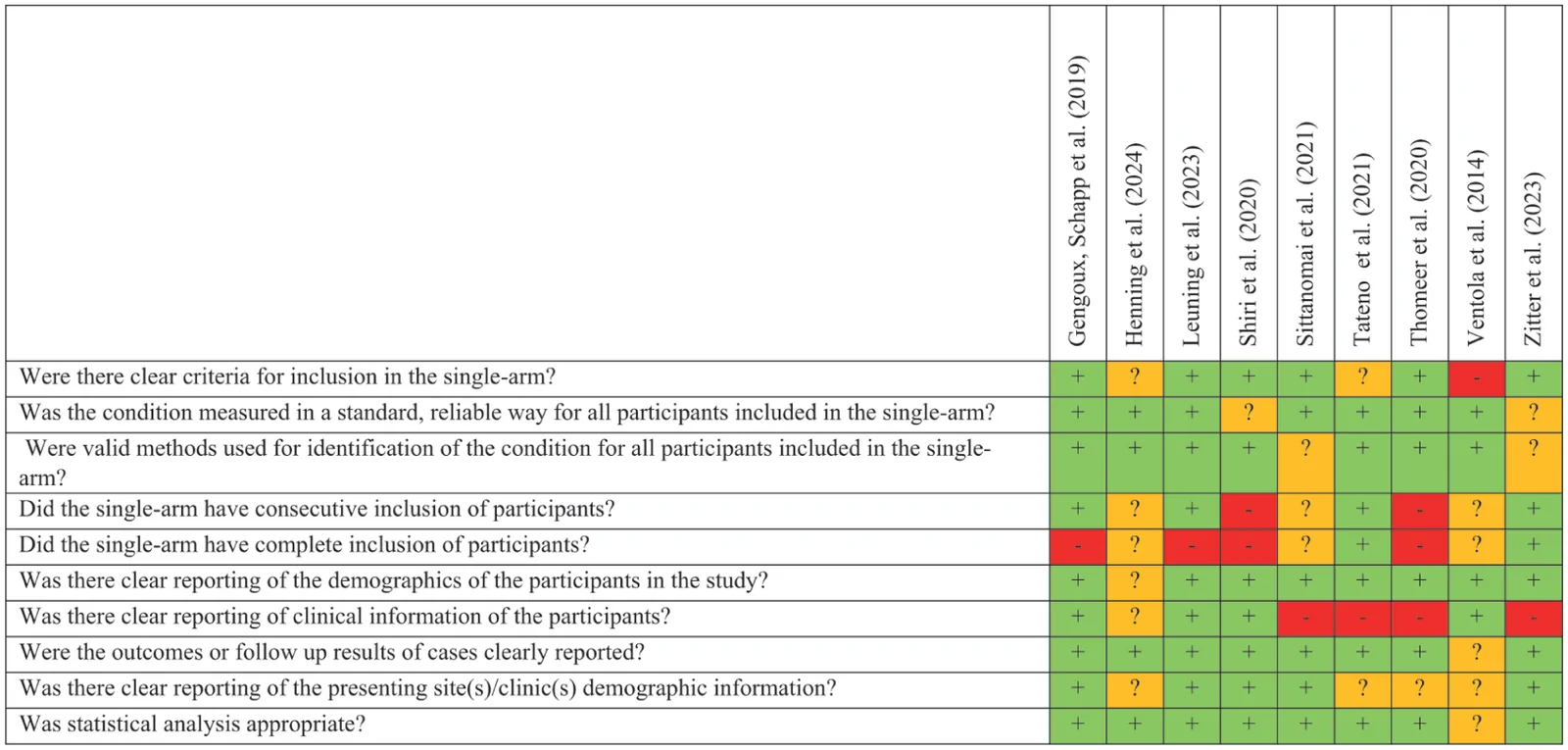
Joanna Briggs Institute critical appraisal tool for single-arm studies, table of results. *Note*. Yes +, Unclear ?, No –.

## Discussion

The present systematic review explored the use of the CGI scale (Guy, 1976) as an outcome measure in psychosocial interventions for autism characteristics.

### Studies’ Characteristics

Most studies applied highly specific inclusion and exclusion criteria, often favouring autistic participants without ID. One study included participants with pervasive developmental disorder-not otherwise specified (Oosterling et al., 2010), published before the *DSM-5* classification (APA, 2013). It is worth adding that comorbidities were inconsistently reported across the studies, limiting the ability to capture participants’ full clinical profiles. The wide age range and heterogeneity of interventions, in terms of focus, duration, mode of delivery, and target outcomes, further complicate comparisons and generalisations within such a heterogeneous clinical presentation and intervention matrix.

### CGI Scale as an Outcome Measure

The CGI scale, originally recommended for pharmacological trials (Aman et al., 2004; White et al., 2007), has increasingly been used in non-pharmacological interventions, particularly the CGI-Improvement scale. Its application, however, has been inconsistent.

In the reviewed studies, many authors evaluated change in global functioning of autistic participants using the CGI scale (as intended), but others focused on narrower domains, such as social communication. It could be argued that change in overall, global impact is too distal from the initial focus of an intervention to be amenable to or even indicate change at all. However, as the CGI scale is fundamentally designed to capture learning or development in areas beyond the direct target of intervention (Sandbank et al., 2021), by shifting the focus to a more specific target, such as social communication skills, the authors this pragmatic adaptation may have compromised the measure’s validity.

Designed as a clinician-rated tool, the CGI was sometimes completed by research staff or directly by parents, with scoring informed by varied sources, including interviews, self-reports, vignettes, and videos. Such variability introduces potential bias, reduces reliability, and limits comparability, particularly in diverse clinical settings. The lack of consensus on appropriate components to inform CGI scoring calls for the development of a more standardised approach to autism-tailored outcome measures. As the psychometric properties of the CGI scale were so rarely reported, echoing prior reviews emphasising the need for validated, reliable measures (McConachie et al., 2015) for this complex and diverse autistic population.

It is also worth noting that the use of a symptom-based global measure such as the CGI in psychosocial or socio-behavioural intervention research presents both opportunities and limitations. On the one hand, tools like the CGI offer simplicity, sensitivity to change, and a broad clinical utility. However, the CGI was developed within a medical model of mental health, where change is often framed in terms of symptom reduction or clinical severity. In the context of autism research, this can be problematic. Psychosocial and behavioural interventions often aim not to decrease or reduce frequency or severity of autism traits per se but to support the development of adaptive skills, well-being, quality of life, and autonomy. Using the CGI in such contexts may unintentionally reinforce deficit-focused perspectives or fail to capture meaningful, person-centred outcomes that fall outside global severity ratings. Attempts to adapt or reinterpret global clinical measures like the CGI to focus on more specific domains, such as social communication, can represent a pragmatic compromise, helping to bridge the gap between the measurable constructs of the medical model and the nuanced, contextual aims of socio-behavioural approaches (Rogers & Vismara, 2008).

### CGI Scale Not Specific to Autism

It is important to note that the CGI scale is not autism-specific. The measure was developed by the National Institute of Mental Health to assess the severity of a patient’s mental illness and the global improvement of a patient in response to treatment (Guy, 1976). However, when performing the literature search for this review, it was noticed that there were several studies that used versions of the CGI scale modified or adapted for autism, such as the Autism Clinical Global Impression – Severity Scale (Kadouri et al., 2007), Clinical Global Impressions Scale – Autism Symptoms (Sandler et al., 1999), Ohio Autism CGI (Butter & Mulick, 2006). These adaptations assess autism-related difficulties, including social communication, repetitive behaviours, hyperactivity, and self-injury. Although valuable for measuring autism-specific change, they differ substantially from the original CGI and were excluded from this review to maintain comparability.

### Clinical Relevance and Future Directions

The reviewed studies demonstrate the CGI scale’s flexibility and clinical utility in capturing overall treatment response in autism research. However, variability in its application, such as differences in rater training, scoring procedures, and adaptation for non-pharmacological contexts, limits cross-study comparability. Clinically, the CGI offers a pragmatic, global assessment of change that can complement autism-specific tools, particularly in multi-component or community-based interventions where nuanced symptom tracking is challenging.

Future research should aim to standardise CGI use within psychosocial intervention trials, including clear guidance on rater training, anchor definitions, scoring methods, and integration with autism-specific outcome measures. A key source of variability in the use of the CGI is inconsistencies in the qualifications of individuals who complete the CGI and the extent of their training. Future studies should consider using independent clinicians or trained evaluators who are blinded to treatment allocation. Standardised training protocols could include reviewing the CGI manual and autism-specific guidance materials, conducting calibration exercises using standardised case vignettes and video-recorded assessments, and achieving acceptable inter-rater agreement before independent scoring. It would be beneficial to determine the amount and type of training required to achieve acceptable reliability and whether raters from different professional backgrounds (e.g., psychologists, speech and language therapists, and research assistants) produce equivalent ratings. Future work should also develop and validate autism-specific anchor descriptions while maintaining the CGI’s original global intent. Consensus methods, such as Delphi panels involving clinicians, researchers, autistic individuals, and family members, may be particularly valuable for establishing person-centred anchor definitions. To establish clearer protocols regarding the information used to generate CGI ratings, a standardised multi-informant approach could be considered, whereby CGI ratings are informed by a predefined combination of sources (e.g., structured caregiver interviews, direct observations, participant self-reports when appropriate, and reviews of intervention goals). Studies should clearly report the data sources contributing to CGI judgments, the time frame over which change is evaluated, and whether ratings refer to global functioning or a specific construct (e.g., social communication). Such adaptations should be explicitly reported as modified applications of the measure. An approach could be adopted in future trials in which CGI ratings are interpreted alongside measures of adaptive functioning, social participation, quality of life, emotional well-being, and intervention-specific targets. Research is needed to determine which combinations of global and autism-specific outcomes provide the most clinically meaningful representation of change and whether CGI improvement corresponds with changes in outcomes valued by autistic individuals and their families. In addition, further validation studies are warranted across broader cognitive and age ranges, including underrepresented groups such as females and autistic individuals with ID, to establish its sensitivity, reliability, and ecological validity in diverse autism populations.

### Strengths and Limitations of the Review

This is the first systematic review examining the CGI scale in psychosocial autism interventions, consolidating existing evidence. A comprehensive search strategy was applied, although grey literature and non-English studies were excluded to ensure rigour of the findings and reduce bias. Several psychosocial interventions were identified in this review that used the CGI-S and/or CGI-I as their outcome measures. The diversity of the interventions, their approaches and aims, or target groups precluded meta-analysis, necessitating a narrative synthesis. Finally, to maintain consistency and accuracy of the presented evidence, the review focused solely on the original version of the CGI scale, however, emerging autism-specific adaptations have also been discussed.

## Conclusion

This review demonstrates that while the CGI scale is widely used to evaluate change in autistic individuals following psychosocial interventions, inconsistent application limits its reliability, validity, and interpretability. Constructs measured, rater roles, scoring procedures, and timing of assessment varied considerably, complicating cross-study comparisons. To improve utility, the CGI should be applied consistently by trained clinicians and alongside other validated assessment tools. Autism-specific adaptations show promise but highlight the need for standardised, reliable outcome measures that capture meaningful, person-centred change beyond global symptom reduction.

## Supplemental Material

sj-docx-1-aut-10.1177_13623613261472939 – Supplemental material for The Use of the Clinical Global Impressions Scale in Psychosocial Interventions for Autism Characteristics: A Systematic Review Supplemental material, sj-docx-1-aut-10.1177_13623613261472939 for The Use of the Clinical Global Impressions Scale in Psychosocial Interventions for Autism Characteristics: A Systematic Review by Magdalena Glod, Victoria Grahame and Jacqui Rodgers in Autism

## Data Availability

The data are available from the corresponding author on reasonable request.

## References

[bibr1-13623613261472939] Aman M. G. Novotny S. Samango-Sprouse C. Lecavalier L. Leonard E. Gadow K. D. King B. H. Pearson D. A. Gernsbacher M. A. Chez M. (2004). Outcome measures for clinical drug trials in autism. CNS Spectrums, 9(1), 36–47. 10.1017/s1092852900008348 PMC4441220 14999174

[bibr2-13623613261472939] American Psychiatric Association. (2000). Diagnostic and statistical manual of mental disorders (4th ed., text rev.). American Psychiatric Publishing.

[bibr3-13623613261472939] American Psychiatric Association, DSM-5 Task Force. (2013). Diagnostic and statistical manual of mental disorders: DSM-5™ (5th ed.). American Psychiatric Publishing. 10.1176/appi.books.9780890425596

[bibr4-13623613261472939] Anello A. Reichenberg A. Luo X. Schmeidler J. Hollander E. Smith C. J. Puleo C. M. Kryzak L. A. Silverman J. M. (2009). Brief report: Parental age and the sex ratio in autism. Journal of Autism and Developmental Disorders, 39(10), 1487–1492. 10.1007/s10803-009-0755-y PMC3056272 19452267

[bibr5-13623613261472939] Bearss K. Johnson C. Smith T. Lecavalier L. Swiezy N. Aman M. McAdam D. B. Butter E. Stillitano C. Minshawi N. Sukhodolsky D. G. Mruzek D. W. Turner K. Neal T. Hallett V. Mulick J. A. Green B. Handen B. Deng Y. Scahill L. (2015). Effect of parent training vs parent education on behavioral problems in children with autism spectrum disorder: A randomized clinical trial. Journal of the American Medical Association, 313(15), 1524–1533. 10.1001/jama.2015.3150 PMC9078140 25898050

[bibr6-13623613261472939] Bishop-Fitzpatrick L. Minshew N. J. Eack S. M. (2013). A systematic review of psychosocial interventions for adults with autism spectrum disorders. Journal of Autism and Developmental Disorders, 43(3), 687–694. 10.1007/s10803-012-1615-8 PMC3508309 22825929

[bibr7-13623613261472939] Brugha T. S. Doos L. Tempier A. Einfeld S. Howlin P. (2015). Outcome measures in intervention trials for adults with autism spectrum disorders; a systematic review of assessments of core autism features and associated emotional and behavioural problems. International Journal of Methods in Psychiatric Research, 24(2), 99–115. 10.1002/mpr.1466 PMC6878529 26077193

[bibr8-13623613261472939] Burns P. B. Rohrich R. J. Chung K. C. (2011). The levels of evidence and their role in evidence-based medicine. Plastic and Reconstructive Surgery, 128(1), 305–310. 10.1097/PRS.0b013e318219c171 PMC3124652 21701348

[bibr9-13623613261472939] Busner J. Targum S. D. (2007). The Clinical Global Impressions Scale: Applying a research tool in clinical practice. Psychiatry, 4(7), 28–37. https://www.ncbi.nlm.nih.gov/pmc/articles/PMC2111444/PMC2880930 20526405

[bibr10-13623613261472939] Butter E. Mulick J. A. (2006). The Ohio Autism Clinical Impressions Scale (OACIS). Institute CsR.

[bibr11-13623613261472939] *de Korte M. W. P. van den Berk-Smeekens I. Buitelaar J. K. Staal W. G. van Dongen-Boomsma M . (2021). Pivotal response treatment for school-aged children and adolescents with autism spectrum disorder: A randomized controlled trial. Journal of Autism and Developmental Disorders, 51(12), 4506–4519. 10.1007/s10803-021-04886-0 33559019

[bibr12-13623613261472939] Delli C. K. S. Polychronopoulou S. A. Kolaitis G. A. Antoniou A. G. (2018). Review of interventions for the management of anxiety symptoms in children with ASD. Neuroscience and Biobehavioral Reviews, 95, 449–463. 10.1016/j.neubiorev.2018.10.023 30395924

[bibr13-13623613261472939] de Nocker Y. L. Toolan C. K . (2023). Using telehealth to provide interventions for children with ASD: A systematic review. Review Journal of Autism and Developmental Disorders, 10(1), 82–112. 10.1007/s40489-021-00278-3 PMC8282771 34306962

[bibr14-13623613261472939] Farmer C. Thurm A. Grant P. (2013). Pharmacotherapy for the core symptoms in autistic disorder: Current status of the research. Drugs, 73(4), 303–314. 10.1007/s40265-013-0021-7 PMC3632301 23504356

[bibr15-13623613261472939] *Freire M. H. André A. M. B. Sampaio R. T. Melo A. (2021). Efficacy of music-centered improvisational music therapy on the treatment of preschool children on the autism spectrum: A controlled study. Revista Brasileira de Musicoterapia, 32, 100–158. 10.51914/brjmt.32.2021.159

[bibr16-13623613261472939] *Gengoux G. W. Abrams D. A. Schuck R. Millan M. E. Libove R. Ardel C. M. Phillips J. M. Fox M. Frazier T. W. Hardan A. Y. (2019). A pivotal response treatment package for children with autism spectrum disorder: An RCT. Pediatrics, 144(3), Article e20190178. 10.1542/peds.2019-0178 PMC6856784 31387868

[bibr17-13623613261472939] *Gengoux G. W. Schapp S. Burton S. Ardel C. M. Libove R. A. Baldi G. Berquist K. L. Phillips J. M. Hardan A. Y. (2019). Effects of a parent-implemented Developmental Reciprocity Treatment Program for children with autism spectrum disorder. Autism: The International Journal of Research and Practice, 23(3), 713–725. 10.1177/1362361318775538 29775078

[bibr18-13623613261472939] *Gengoux G. W. Schwartzman J. M. Millan M. E. Schuck R. K. Ruiz A. A. Weng Y. Long J. Hardan A. Y. (2021). Enhancing social initiations using naturalistic behavioral intervention: Outcomes from a randomized controlled trial for children with autism. Journal of Autism and Developmental Disorders, 51(10), 3547–3563. 10.1007/s10803-020-04787-8 33387236

[bibr19-13623613261472939] *Grahame V. Brett D. Dixon L. McConachie H. Lowry J. Rodgers J. Steen N. Le Couteur A. (2015). Managing repetitive behaviours in young children with autism spectrum disorder (ASD): Pilot randomised controlled trial of a new parent group intervention. Journal of Autism and Developmental Disorders, 45(10), 3168–3182. 10.1007/s10803-015-2474-x 26036646

[bibr20-13623613261472939] Guastella A. J. Gray K. M. Rinehart N. J. Alvares G. A. Tonge B. J. Hickie I. B. Keating C. M. Cacciotti-Saija C. Einfeld S. L. (2015). The effects of a course of intranasal oxytocin on social behaviors in youth diagnosed with autism spectrum disorders: A randomized controlled trial. Journal of Child Psychology and Psychiatry, and Allied Disciplines, 56(4), 444–452. 10.1111/jcpp.12305 25087908

[bibr21-13623613261472939] Guy W. (1976). Clinical global impressions. In ECDEU Assessment Manual for Psychopharmacology, revised (DHEW Publ. No. ADM 76-338, pp. 218–222). National Institute of Mental Health. 10.1037/t48216-000

[bibr22-13623613261472939] Haddaway N. R. Woodcock P. Macura B. Collins A. (2015). Making literature reviews more reliable through application of lessons from systematic reviews. Conservation Biology: The Journal of the Society for Conservation Biology, 29(6), 1596–1605. 10.1111/cobi.12541 26032263

[bibr23-13623613261472939] *Hardan A. Y. Gengoux G. W. Berquist K. L. Libove R. A. Ardel C. M. Phillips J. Frazier T. W. Minjarez M. B. (2015). A randomized controlled trial of Pivotal Response Treatment Group for parents of children with autism. Journal of Child Psychology and Psychiatry, and Allied Disciplines, 56(8), 884–892. 10.1111/jcpp.12354 25346345

[bibr24-13623613261472939] *Henning G. de Oliveira R. R. de Andrade M. T. P. Gallo R. V. Benevides R. R. Gomes R. A. F. Fukue L. E. K. Lima A. V. de Oliveira M. B. B. Z. N. de Oliveira D. A. M. Werpp M. Moraes L. Neto F. L. (2024). Social skills training with a tabletop role-playing game, before and during the pandemic of 2020: In-person and online group sessions. Frontiers in Psychiatry, 14, Article 1276757. 10.3389/fpsyt.2023.1276757 PMC10796767 38250272

[bibr25-13623613261472939] Huffman L. C. Sutcliffe T. L. Tanner I. S. Feldman H. M. (2011). Management of symptoms in children with autism spectrum disorders: A comprehensive review of pharmacologic and complementary-alternative medicine treatments. Journal of Developmental and Behavioral Pediatrics: JDBP, 32(1), 56–68. 10.1097/DBP.0b013e3182040acf 21160435

[bibr26-13623613261472939] Kadouri A. Corruble E. Falissard B. (2007). The improved Clinical Global Impression Scale (iCGI): Development and validation in depression. BMC Psychiatry, 7, Article 7. 10.1186/1471-244X-7-7 PMC1802073 17284321

[bibr27-13623613261472939] Kasari C. Shire S. Shih W. Almirall D. (2021). Getting SMART about social skills interventions for students with ASD in inclusive classrooms. Exceptional Children, 88(1), 26–44. 10.1177/00144029211007148

[bibr28-13623613261472939] *Leuning E. M. van den Berk-Smeekens I. van Dongen-Boomsma M. Staal W. G. (2023). Eye Movement Desensitization and Reprocessing in adolescents with autism; Efficacy on ASD symptoms and stress. Frontiers in Psychiatry, 14, Article 981975. 10.3389/fpsyt.2023.981975 PMC9975260 36873194

[bibr29-13623613261472939] Loomes R. Hull L. Mandy W. P. L. (2017). What is the male-to-female ratio in autism spectrum disorder? A systematic review and meta-analysis. Journal of the American Academy of Child and Adolescent Psychiatry, 56(6), 466–474. 10.1016/j.jaac.2017.03.013 28545751

[bibr30-13623613261472939] Magiati I. Moss J. Charman T. Howlin P. (2011). Patterns of change in children with autism spectrum disorders who received community based comprehensive interventions in their pre-school years: A seven year follow-up study. Research in Autism Spectrum Disorders, 5(3), 1016–1027. 10.1016/j.rasd.2010.11.007

[bibr31-13623613261472939] McConachie H. Parr J. R. Glod M. Hanratty J. Livingstone N. Oono I. P. Robalino S. Baird G. Beresford B. Charman T. Garland D. Green J. Gringras P. Jones G. Law J. Le Couteur A. S. Macdonald G. McColl E. M. Morris C. . . .Williams K. (2015). Systematic review of tools to measure outcomes for young children with autism spectrum disorder. Health Technology Assessment, 19(41), 1–506. 10.3310/hta19410 PMC4781156 26065374

[bibr32-13623613261472939] Munn Z. Barker T. H. Moola S. Tufanaru C. Stern C. McArthur A. Stephenson M. Aromataris E. (2020). Methodological quality of single-arm studies: An introduction to the JBI critical appraisal tool. JBI Evidence Synthesis, 18(10), 2127–2133. 10.11124/JBISRIR-D-19-00099 33038125

[bibr33-13623613261472939] Nashwan A. J. Jaradat J. H. (2023). Streamlining systematic reviews: Harnessing large language models for quality assessment and risk-of-bias evaluation. Cureus, 15(8), Article e43023. 10.7759/cureus.43023 PMC10478591 37674957

[bibr34-13623613261472939] *Oosterling I. Visser J. Swinkels S. Rommelse N. Donders R. Woudenberg T. Roos S. van der Gaag R. J. Buitelaar J. (2010). Randomized controlled trial of the focus parent training for toddlers with autism: 1-year outcome. Journal of Autism and Developmental Disorders, 40(12), 1447–1458. 10.1007/s10803-010-1004-0 PMC2980624 20440639

[bibr35-13623613261472939] Page M. J. McKenzie J. E. Bossuyt P. M. Boutron I. Hoffmann T. C. Mulrow C. D. Shamseer L. Tetzlaff J. M. Akl E. A. Brennan S. E. Chou R. Glanville J. Grimshaw J. M. Hróbjartsson A. Lalu M. M. Li T. Loder E. W. Mayo-Wilson E. McDonald S. . . .Moher D. (2021). The PRISMA 2020 statement: An updated guideline for reporting systematic reviews. British Medical Journal, 372, n71. 10.1136/bmj.n71 PMC8005924 33782057

[bibr36-13623613261472939] Provenzani U. Fusar-Poli L. Brondino N. Damiani S. Vercesi M. Meyer N. Rocchetti M. Politi P. (2020). What are we targeting when we treat autism spectrum disorder? A systematic review of 406 clinical trials. Autism: The International Journal of Research and Practice, 24(2), 274–284. 10.1177/1362361319854641 31269800

[bibr37-13623613261472939] *Rabeyron T. Robledo Del Canto J. P. Carasco E. Bisson V. Bodeau N. Vrait F. X. Berna F. Bonnot O. (2020). A randomized controlled trial of 25 sessions comparing music therapy and music listening for children with autism spectrum disorder. Psychiatry Research, 293, 113377. 10.1016/j.psychres.2020.113377 32798927

[bibr38-13623613261472939] Rogers S. J. Vismara L. A. (2008). Evidence-based comprehensive treatments for early autism. Journal of Clinical Child & Adolescent Psychology, 37(1), 8–38. 10.1080/15374410701817808 PMC2943764 18444052

[bibr39-13623613261472939] Russell G. Stapley S. Newlove-Delgado T. Salmon A. White R. Warren F. Pearson A. Ford T. (2022). Time trends in autism diagnosis over 20 years: A UK population-based cohort study. Journal of Child Psychology and Psychiatry, and Allied Disciplines, 63(6), 674–682. 10.1111/jcpp.13505 34414570

[bibr40-13623613261472939] Sandbank M. Bottema-Beutel K. Crowley S. Cassidy M. Dunham K. Feldman J. I. Crank J. Albarran S. A. Raj S. Mahbub P. Woynaroski T. G. (2020). Project AIM: Autism intervention meta-analysis for studies of young children. Psychological Bulletin, 146(1), 1–29. 10.1037/bul0000215 PMC8783568 31763860

[bibr41-13623613261472939] Sandbank M. Chow J. Bottema-Beutel K. Woynaroski T. (2021). Evaluating evidence-based practice in light of the boundedness and proximity of outcomes: Capturing the scope of change. Autism Research: Official Journal of the International Society for Autism Research, 14(8), 1536–1542. 10.1002/aur.2527 PMC9552805 33943022

[bibr42-13623613261472939] Sandler A. D. Sutton K. A. DeWeese J. Girardi M. A. Sheppard V. Bodfish J. W. (1999). Lack of benefit of a single dose of synthetic human secretin in the treatment of autism and pervasive developmental disorder. The New England Journal of Medicine, 341(24), 1801–1806. 10.1056/NEJM199912093412404 10588965

[bibr43-13623613261472939] *Scahill L. Shillingsburg M. A. Ousley O. Pileggi M. L. Kilbourne R. L. Buckley D. Gillespie S. E. McCracken C. (2022). A randomized trial of direct instruction language for learning in children with autism spectrum disorder. Journal of the American Academy of Child and Adolescent Psychiatry, 61(6), 772–781. 10.1016/j.jaac.2021.11.034 35093490

[bibr44-13623613261472939] Shaw K. A. Williams S. Patrick M. E. Valencia-Prado M. Durkin M. S. Howerton E. M. Ladd-Acosta C. M. Pas E. T. Bakian A. V. Bartholomew P. Nieves-Muñoz N. Sidwell K. Alford A. Bilder D. A. DiRienzo M. Fitzgerald R. T. Furnier S. M. Hudson A. E. Pokoski O. M. . . .Maenner M. J. (2025). Prevalence and early identification of autism spectrum disorder among children aged 4 and 8 years–Autism and developmental disabilities monitoring network, 16 sites, United States, 2022. Morbidity and Mortality Weekly Report. Surveillance Summaries,74(2), 1–22. 10.15585/mmwr.ss7402a1 PMC12011386 40232988

[bibr45-13623613261472939] *Shiri E. Pouretemad H. Fathabadi J. Narimani M. (2020). A pilot study of family-based management of behavioral excesses in young Iranian children with autism spectrum disorder. Asian Journal of Psychiatry, 47, 101845. 10.1016/j.ajp.2019.101845 31683193

[bibr46-13623613261472939] *Shkel J. Geng A. Pilchak E. Millan M. E. Schwartzman J. M. Schuck R. Bundang M. V. Barnowski A. Slap D. M. Stratford S. Hardan A. Y. Phillips J. M. Gengoux G. W. (2025). A pilot randomized controlled trial of motivation-based social skills group treatment with parent training. Journal of Autism and Developmental Disorders, 55(4), 1215–1228. 10.1007/s10803-024-06302-9 PMC11687420 38446265

[bibr47-13623613261472939] *Sittanomai N. Laugeson E. Chantaratin S. Tarugsa J. Sainampran D. Sathirangkul V. Apinantanakul S. Songrujirat N. Boon-yasidhi V. (2021). Social skills training using the Thai version of UCLA PEERS® in Thai adolescents with autism spectrum disorder. Siriraj Medical Journal, 73(7), 471–477. 10.33192/Smj.2021.61

[bibr48-13623613261472939] South M. Rodgers J. Van Hecke A. (2017). Anxiety and ASD: Current progress and ongoing challenges. Journal of Autism and Developmental Disorders, 47(12), 3679–3681. 10.1007/s10803-017-3322-y 28988366

[bibr49-13623613261472939] Sterne J. A. C. Savović J. Page M. J. Elbers R. G. Blencowe N. S. Boutron I. Cates C. J. Cheng H. Y. Corbett M. S. Eldridge S. M. Emberson J. R. Hernán M. A. Hopewell S. Hróbjartsson A. Junqueira D. R. Jüni P. Kirkham J. J. Lasserson T. Li T. . . .Higgins J. P. T. (2019). RoB 2: A revised tool for assessing risk of bias in randomised trials. British Medical Journal, 366, l4898. 10.1136/bmj.l4898 31462531

[bibr50-13623613261472939] Storch E. A. Arnold E. B. Lewin A. B. Nadeau J. M. Jones A. M. De Nadai A. S. Jane Mutch P. Selles R. R. Ung D. Murphy T. K. (2013). The effect of cognitive-behavioral therapy versus treatment as usual for anxiety in children with autism spectrum disorders: A randomized, controlled trial. Journal of the American Academy of Child and Adolescent Psychiatry, 52(2), 132–142.e2. 10.1016/j.jaac.2012.11.007 23357440

[bibr51-13623613261472939] *Tateno Y. Kumagai K. Monden R. Nanba K. Yano A. Shiraishi E. Teo A. R. Tateno M. (2021). The efficacy of Early Start Denver Model intervention in young children with autism spectrum disorder within Japan: A preliminary study. Soa—ch’ongsonyon Chongsin Uihak/Journal of Child & Adolescent Psychiatry, 32(1), 35–40. 10.5765/jkacap.200040 PMC7788666 33424240

[bibr52-13623613261472939] *Thomeer M. L. Lopata C. Rodgers J. D. Donnelly J. P. Jordan A. K. Booth A. J. McDonald C. A. (2020). Feasibility and initial efficacy of a cognitive-behavioral summer treatment for young children with ASD. Journal of Developmental and Physical Disabilities, 32(5), 735–754. 10.1007/s10882-019-09717-w

[bibr53-13623613261472939] Toolan C. Holbrook A. Schlink A. Shire S. Brady N. Kasari C. (2022). Using the Clinical Global Impression scale to assess social communication change in minimally verbal children with autism spectrum disorder. Autism Research: Official Journal of the International Society for Autism Research, 15(2), 284–295. 10.1002/aur.2638 PMC8821201 34800004

[bibr54-13623613261472939] *van den Berk-Smeekens I. de Korte M. W. P. van Dongen-Boomsma M. Oosterling I. J. den Boer J. C. Barakova E. I. Lourens T. Glennon J. C. Staal W. G. Buitelaar J. K . (2022). Pivotal Response Treatment with and without robot-assistance for children with autism: A randomized controlled trial. European Child & Adolescent Psychiatry, 31(12), 1871–1883. 10.1007/s00787-021-01804-8 PMC9663375 34106357

[bibr55-13623613261472939] *Ventola P. Friedman H. E. Anderson L. C. Wolf J. M. Oosting D. Foss-Feig J. McDonald N. Volkmar F. Pelphrey K. A. (2014). Improvements in social and adaptive functioning following short-duration PRT program: A clinical replication. Journal of Autism and Developmental Disorders, 44(11), 2862–2870. 10.1007/s10803-014-2145-3 24915928

[bibr56-13623613261472939] White S. W. Keonig K. Scahill L. (2007). Social skills development in children with autism spectrum disorders: A review of the intervention research. Journal of Autism and Developmental Disorders, 37(10), 1858–1868. 10.1007/s10803-006-0320-x 17195104

[bibr57-13623613261472939] *White S. W. Ollendick T. Albano A. M. Oswald D. Johnson C. Southam-Gerow M. A. Kim I. Scahill L. (2013). Randomized controlled trial: Multimodal Anxiety and Social Skill Intervention for adolescents with autism spectrum disorder. Journal of Autism and Developmental Disorders, 43(2), 382–394. 10.1007/s10803-012-1577-x PMC3494811 22735897

[bibr58-13623613261472939] *White S. W. Richey J. A. Gracanin D. Coffman M. Elias R. LaConte S. Ollendick T. H. (2016). Psychosocial and computer-assisted intervention for college students with autism spectrum disorder: Preliminary support for feasibility. Education and Training in Autism and Developmental Disabilities, 51(3), 307–317. PMC5241080 28111607

[bibr59-13623613261472939] Zaheer J. Links P. S. Liu E. McMain S. (2013). Assessing outcomes in individuals with borderline personality disorder: Psychometric properties of the Clinical Global Impression scale. BMC Psychiatry, 13, Article 4. 10.1186/1471-244X-13-4

[bibr60-13623613261472939] *Zitter A. Rinn H. Szapuova Z. Avila-Pons V. M. Coulter K. L. Stahmer A. C. Robins D. L. Vivanti G. (2023). Does treatment fidelity of the Early Start Denver Model impact skill acquisition in young children with autism? Journal of Autism and Developmental Disorders, 53(4), 1618–1628. 10.1007/s10803-021-05371-4 PMC9160204 34855051

[bibr61-13623613261472939] Zollman C. Vickers A. (1999). What is complementary medicine? British Medical Journal, 319(7211), 693–696. 10.1136/bmj.319.7211.693 PMC1116545 10480829

