# Supplementary material for: The Use of the Clinical Global Impressions Scale in Psychosocial Interventions for Autism Characteristics: A Systematic Review
Source: Autism. 2026 Aug 9;30(10):2481–94. doi: 10.1177/13623613261472939 (PMC13624346; doi:10.1177/13623613261472939)
Supplement: Supplementary file 1 [file sj-docx-1-aut-10.1177_13623613261472939.docx]

**Appendix A**

*Summary of studies*

| Study  *Country* | Intervention name | Intervention aim | Study aim | Design | Sample characteristics | | | | Main findings |
| --- | --- | --- | --- | --- | --- | --- | --- | --- | --- |
|  |  |  |  |  | N | Age  Range  Mean (SD) | Gender | Diagnosis |  |
| de Korte et al. (2021)  *Netherlands* | Pivotal Response Treatment (PRT) | to target “pivotal” areas (i.e. motivation for social contact, self-initiations, self-management, and responding to multiple cues) | to examine the efficacy of clinician-delivered PRT in  school-aged children and adolescents with ASD, integrating  parent training and involvement of teachers | RCT | 44  PRT:22  TAU:22 | 9-15 years old  PRT: 11.87 (1.62)  TAU: 11.70 (2.11) | 31m, 13f  PRT: 16m (72.7%), 6f (27.3 %)  TAU: 15m (68.2%), 7f (31.8 %) | ASD with a total intelligence quotient (TIQ)  of 80 or higher | Positive treatment effects were reported for both groups, larger gains for children and adolescents receiving PRT. |
| Freire et al. (2021)  *Brazil* | Music-centered Improvisational Music Therapy (MCIMT) | to improve communication and interaction | to investigate the effects of MCIMT in the treatment of children with ASD | RCT | 45  Control: 19  MCIMT: 26 | 2-6 years old  Control: 47.32 (11.71) months  MCIMT: 60.38 (9.34) months | Control: 15m (78.9%)  MCIMT: 23m (88.5%) | ASD | Children in the MCIMT group improved on behaviours, communication, and socialization. |
| Gengoux, Abrams et al. (2019)  *USA* | Pivotal Response Treatment Package (PRT-P) | to increase child motivation to  interact | to evaluate a  PRT-P consisting of parent training and clinician-delivered in-home  intervention on the communication skills of children with ASD | RCT | 43  PRT-P: 23  DTG: 20 | 2-5 years old  PRT-P: 49.5 (11.2)  DTG: 47.2 (10.0) | PRT-P: 21m, 2f  DTG: 17m, 3f | ASD and significant language delay | PRT-P was effective for improving child social communication skills. |
| Gengoux, Schapp et al. (2019)  *USA* | Developmental Reciprocity Treatment (DTR) | to build emotional reciprocity | to examine changes in child-, parent- and family-related outcomes following a 12-week DTR programme | Uncontrolled trial | 22 | 2;0-6;11 years old  M=44.6 (12.7) months | 18m (82%), 4f | autism | Improvements were reported in aspects of family empowerment, social quality of life, social deficits, vocabulary,  compulsions, and restricted repetitive behaviours. Reduced severity of social interaction and communication  deficits were reported. |
| Gengoux et al. (2021)  *USA* | Social Initiation Motivation  Intervention (SIMI) | to promote cooperation | to evaluate the effectiveness  of SIMI compared to treatment as usual (TAU) in improving social initiation to peers and social functioning | RCT | 44  SIMI: 22  TAU: 22 | 4-6 years old  SIMI: median 59 months (48-76)  TAU: median 62 months (48-80) | SIMI: 21m (95.5%)  TAU: 21m (95.5%) | ASD without LD (IQ ≥70) | Children who received the SIMI showed gains in  behaviour regulation, social interaction, and joint attention  initiations to peers compared to TAU group. |
| Grahame et al. (2015)  *UK* | Managing Repetitive Behaviours (MRB) | to support parents to recognise, understand and manage RRB in young children with ASD | to explore the utility of the outcome measures  to assess the effects of the intervention on RRB, overall child functioning and parents’ reported self-efficacy | RCT | 45  Immediate: 25  Delayed: 20 | 3-7;11 years old  Immediate: 60.44 (13.44) months  Delayed: 62.75 (16.89) months | Immediate: 24m, 1f  Delayed: 15m, 5f | ASD across a range of abilities | MRB intervention led to greater gains in parent self-efficacy and improvement in overall interaction  between parent and child. |
| Hardan et al. (2015)  *USA* | Pivotal Response Treatment parent training group (PRTG) | to improve functional communication skills in young children with ASD | to examine the  effectiveness of PRT, taught to parents in a group  format, in targeting functional communication deficits  in young children with ASD | RCT | 48  PRTG: 25  PEG: 23 | 2-6 years old  PRTG: 4.1 (1.2)  PEG: 4.1 (1.3) | PRTG: 19m, 6f  PEG: 17m, 6f | autism with delayed communication | PRTG resulted in greater improvement in  frequency of functional utterances compared to PEG.  PRTG participants  demonstrated greater improvement in adaptive communication  skills as reported by parents. |
| Henning et al. (2024)  *Brazil* | tabletop role-playing games (TTRPGs)-based intervention | to improve social skills | to determine the impact of a TTRPG-based intervention in adolescents with ASD | NS | 6 | 13-17 years old | 6m (100%) | ASD (moderate or severe autism excluded) | RPG-based therapeutic intervention improved social skills among autistic adolescents. |
| Leuning et al. (2023)  *Netherlands* | Eye Movement Desensitization and Reprocessing (EMDR) | to increase social skills by reducing stress in individuals with ASD | to investigate whether EMDR on daily experienced stress is effective  in perceived stress, reducing ASD core symptoms and/or improving  global functioning in adolescents with ASD | pre-post-follow up  (uncontrolled) | 21 | 11.9-19.3 years old  15:0 (2:0) years | 12m (57.1%), 9f (42.9%) | ASD and a full scale IQ ≥ 80 | Partial effect of EMDR were reported in autistic adolescents on ASD symptoms. EMDR significantly reduced perceived stress and improved global clinical functioning. |
| Oosterling et al. (2010)  *The Netherlands* | Focus parent training | to stimulate joint attention and language skills | to establish whether some children benefited more than others, based on their developmental potential | RCT | 67  Experimental group:36  Control group:31 | Toddlers  Experimental group:35.2 (5.5) months  Control group:33.3 (6.4) months | Experimental group: 75%m  Control group:80.6%m | autism and a developmental age of at least  12 months or PDD-NOS  and a developmental age of at least  12 months and a Developmental Quotient (DQ) < 80 | No expected changes or improvements reported. |
| Rabeyron et al. (2020)  *France* | Music therapy (MT) | to promote health and psychological change (improvement of specific autistic symptoms) | to assess  the efficacy of music therapy on autistic symptoms of children with ASD | RCT | 37 (36 completed)  Music Therapy: 19  Music Listening: 17 | 4-7 years old | 31 boys and 5  girls | ASD | Music therapy was overall more efficient than simply listening to music for autistic  children. |
| Scahill et al. (2022)  *USA* | Direct  Instruction Language for Learning (DI) | to improve expressive and receptive language skill development in children | to evaluate the efficacy of Direct Instruction Language for Learning in preschool  and young school-age children with ASD and moderate language delay | RCT | 83  DI + TAU: 42  TAU: 41 | 4-7 years 11 months  DI + TAU: 5.63 (1.13) years  TAU: 5.62 (1.00) years | DI + TAU: 36m (85.7%)  TAU: 35m (85.4%) | ASD and moderate language delay | No expected changes or improvements reported on primary outcome measure. DI+TAU was superior to  TAU on the CGI-I. |
| Shiri et al. (2020)  *Iran* | Family-based Management of Behavioral Excesses of Autism Program (FMBEAP) | to improve  excessive behaviours in young Iranian children with ASD | to introduce an early bird home-based intensive intervention  to assess the feasibility and efficacy of the program on behavioral excesses | quasi-experimental, pre, post and follows up assessment | 17 | 24–48 months,  35.29 (7.15) months | 15m (88.23%)  2f (11.76%) | ASD | Some evidence was reported for feasibility, acceptability  and the efficacy of FMBEAP. |
| Shkel et al. (2025)  *USA* | Systematic Use of Cooperative Contingencies to Enhance Social Success (SUCCESS) | to improve social skills | to evaluate the effective­ness of SUCCESS compared to the Treatment As Usual group (TAU) | RCT | 25 | 4-6 years old  62 (8.2) months | 18m, 7f | ASD without intellectual disability (IQ ≥ 70) | Preliminary support for the efficacy of the SUCCESS was reported. |
| Sittanomai et al. (2021)  *Thailand* | University of California Los Angeles (UCLA)  Program for the Education and Enrichment of Relational Skills (PEERS®) (Thai version) | to teach social skills to adolescents with ASD | to assess the feasibility and effectiveness of this modified (Thai) version of UCLA PEERS® in Thai adolescents with ASD | NS | 12 | 11-19 years old  14.8 (1.99) years | 10m (83.8%) | ASD and an intelligence quotient (IQ) above the intellectual disability level (>70) | Social skills improvement was reported after the intervention. |
| Tateno et al. (2021)  *Japan* | Early Start Denver Model  (ESDM) | to reduce the severity of ASD diagnosis | to investigate the clinical efficacy of ESDM intervention in young children with ASD in Japan | NS | 27 | 15-40 months old  29.4 (6.4) months old | 18m, 9f | ASD | ESDM intervention was reported to reduce the severity of social impairments and language delay. |
| Thomeer et al. (2020)  *USA* | cognitive-behavioral  summer treatment (summerMAX^yc^) | to increase social/social-communication skills, face-emotion  decoding skills, and interest expansion | to further assess the feasibility and initial outcomes of the summerMAX^yc^ | Open trial, non-randomised | 12 | 4-6 years old  5.86 (0.75) | 9m (75.0%)  3f (25.0%) | HFASD  (ASD and  abbreviated IQ >70 and expressive or receptive language score > 80 | Significant gains were reported on the children’s social performance and secondary measures of social/social-communication skills and ASD symptoms, and a range of adaptive behaviours. |
| van den Berk‑Smeekens et al. (2022)  *The Netherlands* | Pivotal Response Treatment (PRT) and robot-assisted PRT | to focus on improving social communication skills | to assess the efficacy of PRT (with and  without robot-assistance) compared to TAU in (1) improving  general social-communicative skills and (2) improving  clinical global functioning | RCT | 83  PRT: 25  PRT+robot: 25  TAU: 23 | 3–8 years  PRT: 6.43 (1.71)  PRT+robot: 6.18 (1.31) TAU: 6.09 (1.30) | PRT: 22m (88%)  PRT+robot : 20m (80%)  TAU: 19mde (82.61%) | ASD  (TIQ>70) | All groups were reported to improve on general social-communicative skills, the largest improvements were reported for children  who received robot-assisted PRT. |
| Ventola et al. (2014)  *USA* | Pivotal Response Treatment (PRT) | to improve social  communication skills by addressing core deficits in social motivation or pivotal responses | to examine the efficacy of a short-duration  (16-week) PRT intervention for ten cognitively able preschool-aged children with ASD using a clinical  replication design | Clinical replication | 10 | 4.5–6.11 years  5.4 (0.71) years | 2f, 8m | ASD  (cognitively-able) | PRT was reported to be effective  in improving social and communication skills in autistic children. |
| White et al. (2013)  *USA* | Multimodal  Anxiety and Social Skills Intervention (MASSI) | to target concurrent anxiety and social disability in adolescents with ASD via individual  therapy, group social skills training, and parent  coaching | to evaluate  the feasibility and preliminary outcomes of the  MASSI  program | RCT | 30 | Adolescents, 175 months (15 years)  MASSI: 170 (14 years) WL: 180 (15 years) | 23m (77 %)  MASSI: 11m (73 %)  WL: 12m (80 %) | ASD and at least one of  four anxiety disorders, (Social/Specific Phobia,  Separation or  Generalized Anxiety Disorder), a current verbal IQ ≥70 and no previous diagnosis  of intellectual disability | Feasibility of the MASSI for social deficits and anxiety in autistic adolescents  was reported. |
| White et al. (2016)  *USA* | College and Living Success (CLS)  Brain-Computer Interface for ASD (BCI- ASD) | CLS: to improve social competence and self-regulation  BCI-ASD: to improve social competence, with emphasis on emotion recognition | to examine the feasibility of each intervention model, and to examine preliminary behavioural outcomes | RCT | 8 | 18-25 years old  CLS: 20.25 (1.71) years  BCI-ASD: 20.75 (1.71) years | 5m, 3f | ASD | Lack of firm conclusions about the preliminary efficacy of either program was reported. |
| Zitter et al. (2023)  *USA* | Group–Early  Start Denver Model (G-ESDM) | to target individual goals within group-based cooperative learning experiences | to examine the feasibility of implementing the  G-ESDM in a community early child education setting serving under-resourced communities | NS | 5 | 32 to 41 months | 5m | ASD | Most children were reported to show improvements across various domains of functioning. |

*Note:* m – male, f – female, RCT – randomised controlled trial, TAU – treatment as usual, ASD – autism spectrum disorder, DTG - delayed treatment group, ADHD – attention deficit hyperactivity disorder, RRB - restricted and repetitive behaviours, PEG - psychoeducation group, SMART - sequential multiple-assignment randomized trial, RR - Remaking Recess, CS - classroom supports, NS – not stated, HFASD – high-functioning children with autism spectrum disorder, WL – Wait-List, PDD-NOS - pervasive developmental disorder-not otherwise specified, TIQ - total intelligence quotient

**Appendix B**

*CGI scale data extraction form*

| Study | Intervention name  Duration of intervention | CGI version | | CGI as an outcome measure | | | | CGI as an assessment of? | Components considered to inform the CGI scoring | Rater | Blinding | Inter-rater  reliability reported | CGI timepoints | CGI scoring  7-point scale | CGI interpretation |
| --- | --- | --- | --- | --- | --- | --- | --- | --- | --- | --- | --- | --- | --- | --- | --- |
|  |  | CGI-S | CGI-I | baseline | primary | secondary | not specified |  |  |  |  |  |  |  |  |
| Randomised controlled trials (RCTs) | | | | | | | | | | | | | | |  |
| de Korte et al. (2021) | Pivotal Response Treatment (PRT)  12 (+8) weeks | x | **X** | x |  | **X** |  | clinical global functioning | information about clinical status of overall functioning, symptoms, and well-being in major areas of the participants life provided  by the coordinating therapist | two experienced child- and adolescent psychiatrists | yes | no | week 10 (to determine if extension of 8 weeks was necessary  after 12 weeks intervention), week 20 and week 28 | yes | Clinical responders – rating of very much improved (1) or much improved (2) |
| Freire et al. (2021) | Music-centered Improvisational Music Therapy (MCIMT)  16 weeks | **X** | **X** |  |  |  | **X** | general behaviour and performance | interview with parents | NR | NR | no | baseline (T1), week 16 (T2), follow-up after 2 months (T3) for intervention group only | NR | NR |
| Gengoux, Abrams et al. (2019) | Pivotal Response Treatment Package (PRT-P)  24 weeks | **X** | **X** |  |  | **X** |  | social communication skills | NR | senior investigator | yes | NA  1 rater | baseline,  week 24 | NR | NR |
| Gengoux et al. (2021) | Social Initation Motivation  Intervention (SIMI)  8 weeks |  | **X** |  |  | **X** |  | social communication  improvement | reviewed videos collected at baseline and week 8 and telephone interviews with each parent focusing on changes observed over the 8-week period | clinician not involved in other aspects of the study | yes | NA  1 rater | baseline, 8 weeks | yes | NR |
| Grahame et al. (2015) | Managing Repetitive Behaviours (MRB)  8 weeks |  | **X** |  | **X** |  |  | global improvement in overall interaction between parent  and child and how much the child’s RRB had changed over  the 24 weeks since baseline | information from all time points (e.g.  questionnaires, ADOS 2 videos, target behaviour vignettes and videos of parent child interactions) | independent panel of expert ASD clinicians | yes | no | baseline, week 10, 18 and 24 | yes | Improvement – rating of very much improved (1) or much improved (2) |
| Hardan et al. (2015) | Pivotal Response Treatment parent training group (PRTG)  12 weeks | **X** | **X** |  |  | **X** |  | social and  communication skills | NR | psychologist | yes | NA  1 rater | baseline, week 6, and week 12 | NR | NR |
| Oosterling et al. (2010) | Focus parent training  12 months |  | **X** |  |  | **X** |  | clinical global improvement | comprehensive assessment reports | psychologist | yes | NA  1 rater | endpoint | yes | NR |
| Rabeyron et al. (2020) | Music therapy (MT)  8 months |  | **X** |  | **X** |  |  | severity | NR | two trained clinical psychologists | yes | no | t0 (start of the study) and t1 (after 25  sessions) | partially  (reported scoring for the CGI-S, but used the CGI-I) | Improvement clinically meaningful - a decrease of at least 2 points at the CGI |
| Scahill et al. (2022) | Direct  Instruction Language for Learning (DI)  24 weeks |  | **X** |  |  | **X** |  | overall improvement  from baseline | Aberrant Behavior Checklist, Parent Target Problem narratives and, at Week 24, CELF scores | independent evaluator (IE) | yes | NA  1 rater | weeks 8, 12, 16 and 24 during the randomized trial and at week-36 and week-48 post-treatment follow up | yes | Positive response - rating of very much improved (1) or much improved (2)  Negative response - all  other scores |
| Shkel et al. (2025) | Systematic Use of Cooperative Contingencies to Enhance Social Success (SUCCESS)  12 weeks |  | **X** |  |  |  | **X** | social communication improvement | review of baseline and week 12 videos and parent interviews (changes observed over the 12-week period) | clinician not involved in other aspects of the study | yes | NA  1 rater | week 12 | yes | NR |
| van den Berk‑Smeekens et al. (2022) | Pivotal Response Treatment (PRT) and robot-assisted PRT  20 weeks |  | **X** |  | **X** |  |  | change in clinical global functioning | information  about the clinical status of functioning, symptoms,  and well-being in major areas of the participants life provided  by the coordinating therapist | experienced child- and adolescent psychiatrists | yes | NR | week 10, endpoint, and follow-up | yes | Clinical responder –  rating of very much improved (1) or much improved (2) |
| White et al. (2013) | Multimodal  Anxiety and Social Skills Intervention (MASSI)  14 weeks |  | **X** |  |  | **X** |  | global functioning  (including functioning in school, activities of daily living, social activities, and home) | parent and adolescent interviews at baseline to assess severity and at the endpoint to assess improvement | independent  evaluators (IEs) | yes | no, but all IEs were trained to a pre-set reliability standard | baseline and endpoint | yes | Responder - rating of very much improved (1) or much improved (2)  Non-responder - all other scores |
| White et al. (2016) | College and Living Success (CLS)  Brain-Computer Interface for ASD (BCI- ASD)  10-14 weeks |  | **X** |  |  |  | **X** | overall functioning | all available data, including self-report measures, interviews, and observations | clinician | yes | NA  1 rater  but the assessor was trained to a pre-set reliability standard | baseline and endpoint | yes | Treatment responders - ratings of 1, 2, and 3  Non-responders - ratings of 4 or higher |
| Single-arm (pre- post- design) | | | | | | | | | | | | | | |  |
| Gengoux, Schapp et al. (2019) | Developmental Reciprocity Treatment (DTR)  12 weeks | **X** | **X** |  |  |  | **X** | social and communication skills | parent interviews | clinician with  extensive experience in the assessment and treatment of  children with developmental disabilities | no | NA  1 rater | baseline, week 12 | yes | NR |
| Henning et al. (2024) | tabletop role-playing games (TTRPGs)-based intervention  12 sessions over a 12-month period | **X** | **X** |  |  |  | **X** | severity of the social skills deficits exhibited | NR | Parents/carers | no | no | each session -CGI-S  endpoint – CGI-I | yes | NR |
| Leuning et al. (2023) | Eye Movement Desensitization and Reprocessing (EMDR)  10 weeks | **X** | **X** | x |  | **X** |  | clinical global functioning | information  about the clinical status of functioning, symptoms, and well-being in major areas of the participants life provided by the coordinating therapist of the participant | experienced child psychiatrists | yes | no | baseline - CGI-S  endpoint  and follow-up - CGI-I | yes | Clinical responder - rating of very much improved (1) or much improved (2) |
| Shiri et al. (2020) | Family-based Management of Behavioral Excesses of Autism Program (FMBEAP)  10 weeks |  | **X** |  |  |  | **X** | global improvement or change | all available  information (RBS-R, VABS and video-recorded observation method in  the clinic) | independent evaluator | yes | NA  1 rater | endpoint | yes | Responder – rating of very much improved (1) or much improved (2) |
| Sittanomai et al. (2021) | University of California Los Angeles (UCLA)  Program for the Education and Enrichment of Relational Skills (PEERS®) (Thai version)  10 weeks |  | **X** |  |  |  | **X** | global improvement in the patient’s illness | NR | psychiatrists | no | no | endpoint | yes | NR |
| Tateno et al. (2021) | Early Start Denver Model  (ESDM)  at least 12 weeks | **X** |  |  |  |  | **X** | overall severity of ASD symptoms, behavior, and function | NR | two of team members – consensus score  (CGI-S rater),  but also parents/caregivers (CGI-S parent) | NR | no | pre- and post-treatment | yes | NR |
| Thomeer et al. (2020) | cognitive-behavioral  summer treatment (summerMAXyc)  5 weeks |  | **X** |  | **X** |  |  | child’s overall (global)  social impairment/performance | video recording of children prior to treatment and post-treatment during 20-min unstructured free-play sessions with two other autistic children that received the treatment | two independent raters | yes | yes, absolute agreement between  the two raters for each video was 83.3% on the level of social  impairment and 100% on the improvement rating | pre- and post-treatment | yes | Responder – rating of very much improved (1) or much improved (2)  Non-responder – all other scores |
| Ventola et al. (2014) | Pivotal Response Treatment (PRT)  16 weeks |  | **X** |  |  |  | **X** | overall clinical effect | independent review of all available records | two independent raters, doctoral-level clinicians who specialised in  work with children with ASD with many years of  experience with this population, trained in the use of the CGI in clinical trials | yes | no, but the reliability training described | pre- and post-treatment | partially  (redefines the anchor points e.g., minimally improved was defined as ‘‘clear indication that behavior or functioning has improved beyond day-to-day variation’’) | NR |
| Zitter et al. (2023) | Group–Early  Start Denver Model (G-ESDM)  6 months |  | **X** |  |  |  | **X** | change in the children’s global functioning | review of available records (i.e. MSEL and VABS-3 scores) | two licensed psychologists who specialised in working with children with ASD | yes | no, but the reliability training described | pre- and post-treatment | yes | NR |

*Note:* **X** – CGI scale used as an outcome measure, x – CGI scale used as a baseline characterisation measure, NA – not applicable, NR – not reported

**Appendix C**

*Selected statistics and effect sizes for the CGI scale*

| Study | Intervention name  and duration | Power calculation | Power achieved? | Significant CGI | | Effect size Cohen’s *d* | | | | Other statistics/comments | | | |
| --- | --- | --- | --- | --- | --- | --- | --- | --- | --- | --- | --- | --- | --- |
|  |  |  |  | CGI-S | CGI-I | CGI-S | CI | CGI-I | CI |  | | | |
| Randomised controlled trials (RCTs) | | | | | | | | | | | | | |
| de Korte et al. (2021) | Pivotal Response Treatment (PRT)  12 (+8) weeks | yes | yes |  | no  *p*>0.05 |  |  | Calculated  Week 12: 0.90  Week 20: 0.83  Follow-up: 1.06 | 0.27 to 1.51  0.20 to 1.43 0.41 to 1.67 |  | | | |
| Freire et al. (2021) | Music-centered Improvisational Music Therapy (MCIMT)  16 weeks | no | NA | yes  *p*=.011 | yes  *p*=.001 | Calculated  0.88 | 0.25 to 1.49 | Calculated  1.14 | 0.49 to 1.76 | When comparing endpoint and follow-up in the intervention group, the children worsened on the CGI-I (large effect size *d*=1.691, *p*<0.001) | | | |
| Gengoux, Abrams et al. (2019) | Pivotal Response Treatment Package (PRT-P)  24 weeks | yes | no | yes  *p*=.019 | yes  p<.001 | 0.44 | -0.18 to 1.04 | 1.06 | 0.40 to 1.68 |  | | | |
| Gengoux et al. (2021) | Social Initation Motivation  Intervention (SIMI)  8 weeks | no | NA |  | yes  *p*=.028 |  |  |  |  | SIMI group  much improved: 7/22 (32%)  minimally improved: 10/22 (46%)  no change: 5/22 (23%) | | TAU  much improved: 3/20 (15%)  minimally improved: 4/20 (20%)  no change: 13/20 (65%) | |
| Grahame et al. (2015) | Managing Repetitive Behaviours (MRB)  8 weeks | no | NA |  | yes  *p*=.05 |  |  |  |  | immediate intervention group (IG)  very much improved/much improved: 7/23 (30%)  minimally improved: 6/23 (26 %)  no change: 10/23 (44 %) | | delayed intervention group (DG)  very much improved/  much improved: 1/20 (5 %)  minimally improved: 4/20 (20 %)  no change: 15/20 (75 %) | |
| Hardan et al. (2015) | Pivotal Response Treatment parent training group (PRTG)  12 weeks | yes | no | yes  *p*=.003 | yes  *p*=.001 | 0.47 | -0.12 to 1.04 | 1.14 | 0.51 to 1.74 |  | | | |
| Oosterling et al. (2010) | Focus parent training  12 months | yes | no |  | no  *p*=0.05 |  |  |  |  | experimental group  much improvement: 21/36 (57%)  minimal or no improvement: 15/36 (43%) | | control group  much improvement: 16/31 (52%)  minimal or no improvement: 15/31 (48%) | |
| Rabeyron et al. (2020) | Music therapy (MT)  8 months | no | NA |  | yes  *p*=.005 |  |  |  |  | MT group  improvement of at least 1 point: 17/19 (89.5%) | | ML group  improvement of at least 1 point: 14/17 (82.4%) | |
| Scahill et al. (2022) | Direct  Instruction Language for Learning (DI)  24 weeks | yes | Partially  *post-hoc* power calculation that estimated 72% power to detect an effect size of  0.6, and 80% power to detect an effect size of 0.66. |  | yes |  |  |  |  | DI+TAU  much improved or very much improved: 23/42 (54.8%) | | TAU  much improved or very much improved: 9/41 (21.9%) | |
| Shkel et al. (2025) | Systematic Use of Cooperative Contingencies to Enhance Social Success (SUCCESS)  12 weeks | no | NA |  | yes  *U*= 23, *p*= .013 |  |  |  |  | SUCCESS group  much improved: 7/11 (64%)  mini­mally improved: 2/11 (18%)  no change: 2/11 (18%) | | TAU  much improved: 0/11 (0%)  minimally improved: 6/11 (55%)  no change: 5/11 (45%) | |
| van den Berk‑Smeekens et al. (2022) | Pivotal Response Treatment (PRT) and robot-assisted PRT  20 weeks | no | NA |  | no |  |  |  |  | PRT Endpoint 25  Clinical responders: 11/25 (44%)  PRT  Follow-up  Clinical responders: 12/25 (48%) | PRT+robot Endpoint 25  Clinical responders: 17/25 (66.7%)  PRT+robot Follow-up  Clinical responders: 18/25 (70.8%) | | TAU Endpoint 23  Clinical responders: 8/23 (36.4%)  TAU  Follow-up  Clinical responders: 10/23 (45.5%) |
| White et al. (2013) | Multimodal  Anxiety and Social Skills Intervention (MASSI)  14 weeks | yes | no |  | no |  |  |  |  | MASSI  Responders: 6/15 (40 %) | | WL  Responders: 3/15 (20 %) | |
| White et al. (2016) | College and Living Success (CLS)  Brain-Computer Interface for ASD (BCI- ASD)  10-14 weeks | no | NA |  | no |  |  |  |  | CLS  Responders: 2/3 (66.6%) | | BCI-ASD  Responders: 2/3 (66.6%) | |
| Single-arm (pre- post- design) | | | | | | | | | | | | | |
| Gengoux, Schapp et al. (2019) | Developmental Reciprocity Treatment (DTR)  12 weeks |  |  | yes  *p*=.005 | Improvements reported | 0.60 | -0.18 to 1.02 |  |  | DTR  much improved: 9/22 (41%)  mini­mally improved: 12/22 (54.5%)  no change: 1/22 (4.5%) | | | |
| Henning et al. (2024) | tabletop role-playing games (TTRPGs)-based intervention  12 months |  |  | NR | NR |  |  |  |  | NR | | | |
| Leuning et al. (2023) | Eye Movement Desensitization and Reprocessing (EMDR)  10 weeks |  |  | yes  *p*=.003  baseline to endpoint  *p*=.016 baseline to follow-up | Improvements reported | Calculated  baseline to endpoint 1.18  baseline to follow-up 0.96 | 0.50 to 1.81  0.31 to 1.58 |  |  | EMDR  clinical significant response at endpoint: 11/21 (52.4%)  clinical significant response at follow-up: 5/21 (23.8%) | | | |
| Shiri et al. (2020) | Family-based Management of Behavioral Excesses of Autism Program (FMBEAP)  10 weeks | no | NA |  | NR |  |  |  |  | FMBEAP  very much improved: 8/17 (47%)  much improved: 7/17 (41%)  mini­mally improved or no change: 2/17 (12%) | | | |
| Sittanomai et al. (2021) | University of California Los Angeles (UCLA)  Program for the Education and Enrichment of Relational Skills (PEERS®) (Thai version)  10 weeks |  |  |  | NR |  |  |  |  | UCLA PEERS®  very much improved or much improved: 6/12 (50%) | | | |
| Tateno et al. (2021) | Early Start Denver Model  (ESDM)  12+ weeks |  |  | yes  *p*＜.001  both CGI-S rater and parent |  | calculated  CGI-S rater 1.70  CGI-S parent 1.51 | 1.05 to 2.29  0.88 to 2.09 |  |  |  | | | |
| Thomeer et al. (2020) | cognitive-behavioral  summer treatment (summerMAXyc)  5 weeks |  |  |  | yes  *p*= .02 |  |  | 1.76 |  | summerMAXyc  very much improved or much improved: 10/12 (83.3%) | | | |
| Ventola et al. (2014) | Pivotal Response Treatment (PRT)  4 months |  |  |  | NR |  |  |  |  | PRT *n*=10  All of children were rated as at least minimally improved by one of the raters. | | | |
| Zitter et al. (2023) | Group–Early  Start Denver Model (G-ESDM)  6 months |  |  |  | NR |  |  |  |  | G-ESDM *n*=5  All, but one, children were rated at least “minimally improved” by both raters. | | | |

*Note:* NA – not applicable, NR – not reported
